# Cryoablation Activates the cGAS–STING‐CXCL10 Axis in Macrophages to Enhance Anti‐Tumor Immunity in NSCLC

**DOI:** 10.1002/advs.202521931

**Published:** 2026-03-12

**Authors:** Xinxin Zhi, Zhengcao Xing, Libo Luo, Jiale Wang, Xinyu Liu, Jia Yu, Jizhong Yin, Bin Chen, Yiwei Liu, Hui Sun, Guanghui Gao, Lei Wang, Xiaoxia Chen, Fei Li, Hu Ma, Lin Wang, Shuo Yang, Shengxiang Ren

**Affiliations:** ^1^ Department of Medical Oncology, Shanghai Pulmonary Hospital, School of Medicine Tongji University Shanghai China; ^2^ Department of Pathology and Frontier Innovation Center, School of Basic Medical Sciences Fudan University Shanghai China; ^3^ Department of Oncology The Second Affiliated Hospital of Zunyi Medical University Zunyi China

**Keywords:** cGAS‐STING signalling, cryoablation, CXCL10^+^ macrophages, NSCLC

## Abstract

Local ablative therapy has emerged as an essential treatment for patients with non‐small cell lung cancer (NSCLC). Whether cryoablation is superior to thermal ablation in the era of immunotherapy and the related mechanism remains undefined. We first observed superior progression‐free survival with cryoablation compared with thermal ablation in patients with oligoresidual disease after immunotherapy. Single‐cell RNA sequencing of human peripheral blood monocyte cells and mouse tumors showed that cryoablation combined with anti‐PD‐1 expanded more CXCL10^+^ macrophages than thermal ablation combination. CXCR3 blockade and inhibition of T cells egressing from draining lymph nodes abolished the systemic anti‐tumor efficacy. Mechanistically, tumor DNA released by cryoablation was taken up by macrophages, activating the cGAS–STING signaling pathway, increasing the pool of CXCL10^+^ macrophages and CXCL10 secretion. Our study demonstrated that CXCL10^+^ macrophages and the CXCR3^+^ T cells were critical mediators of the systemic anti‐tumor immunity induced by cryoablation in advanced NSCLC.

## Graphical abstract

Tumor dsDNA released by cryoablation is taken up by macrophages, activating the cGAS‐STING signaling pathway. This leads to an expansion of the CXCL10^+^ macrophage pool and increased secretion of CXCL10, which in turn recruits CXCR3^+^ T cells from draining lymph nodes into the tumor microenvironment to exert anti‐tumor effects.

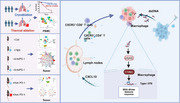

## 1 Introduction

Immunotherapy has become the backbone for patients with driver gene‐negative non‐small cell lung cancer (NSCLC) [1]. The observed efficacy benefit of various combination therapies is propelling immunotherapy into a new phase focusing on combination approaches [2, 3, 4]. Recently, computed tomography (CT)‐guided percutaneous ablation has become an increasingly used modality for oligo‐progressive or residual NSCLC [5, 6]. As a novel treatment modality, ablation can not only eliminate residual subclones that become the primary source of disease progression but also effectively activate the anti‐tumor immune response, synergistically enhancing the efficacy of immunotherapy [7, 8].

Ablation includes thermal ablation and cryoablation. Thermal ablation employs microwave or radiofrequency to heat tumor tissues to over 60°C, resulting in coagulative necrosis [7]. Multiple studies have confirmed that thermal ablation can promote dendritic cells (DCs) antigen presentation [9], suppress regulatory T cells (Tregs) differentiation [10], enhance the infiltration of CD8^+^ T cells and natural killer cells (NKs) [11], and increase secretion of chemokines such as IL‐6 and IL‐10 [12]. Increasing evidence has found that heat shock protein was the key molecule in stimulating the antitumor immune response after thermal ablation [13, 14]. These effects lead to immune activation both systemically and within the tumor microenvironment, thereby producing synergistic antitumor responses.

Cryoablation, in contrast, induces liquefactive necrosis and protein denaturation of cancer cells through rapid freezing and slow thawing, thus better preserving the immunogenicity of tumor antigens and facilitating the release of damage‐associated molecular patterns (DAMPs) [8, 15]. Therefore, when combined with immunotherapy, cryoablation may yield superior clinical outcomes and immune cell infiltration than thermal ablation. Several studies have investigated the immunomodulatory mechanisms of cryoablation and established the roles of Tregs [16], DCs [17, 18], and CD8^+^ T cells [19]. Subgroup analysis from our BOOSTER study found cryoablation plus ICIs yielded prolonged PFS compared to those with thermal ablation and with a trend toward prolonged OS [20]. However, compared to thermal ablation, the critical molecular mechanisms underlying the superior immunostimulatory effects of cryoablation and the role of macrophages remain unclear.

Aiming to investigate the anti‐tumor effects of cryoablation and its potential mechanisms, we retrospectively collected the data of combination ablation with immunotherapy in Shanghai Pulmonary Hospital, Tongji University. We further delineated the immune microenvironmental changes induced by cryoablation and thermal ablation by collecting peripheral blood mononuclear cells (PBMCs) of ablated patients and mouse models. Moreover, we investigated the synergistic immune mechanisms of macrophages and T cells in cryoablation and identified key molecules and corresponding signaling pathways.

## 2 Results

### 2.1 Cryoablation Increased the Proportion of Macrophages in PBMC in Patients

**FIGURE 1:**
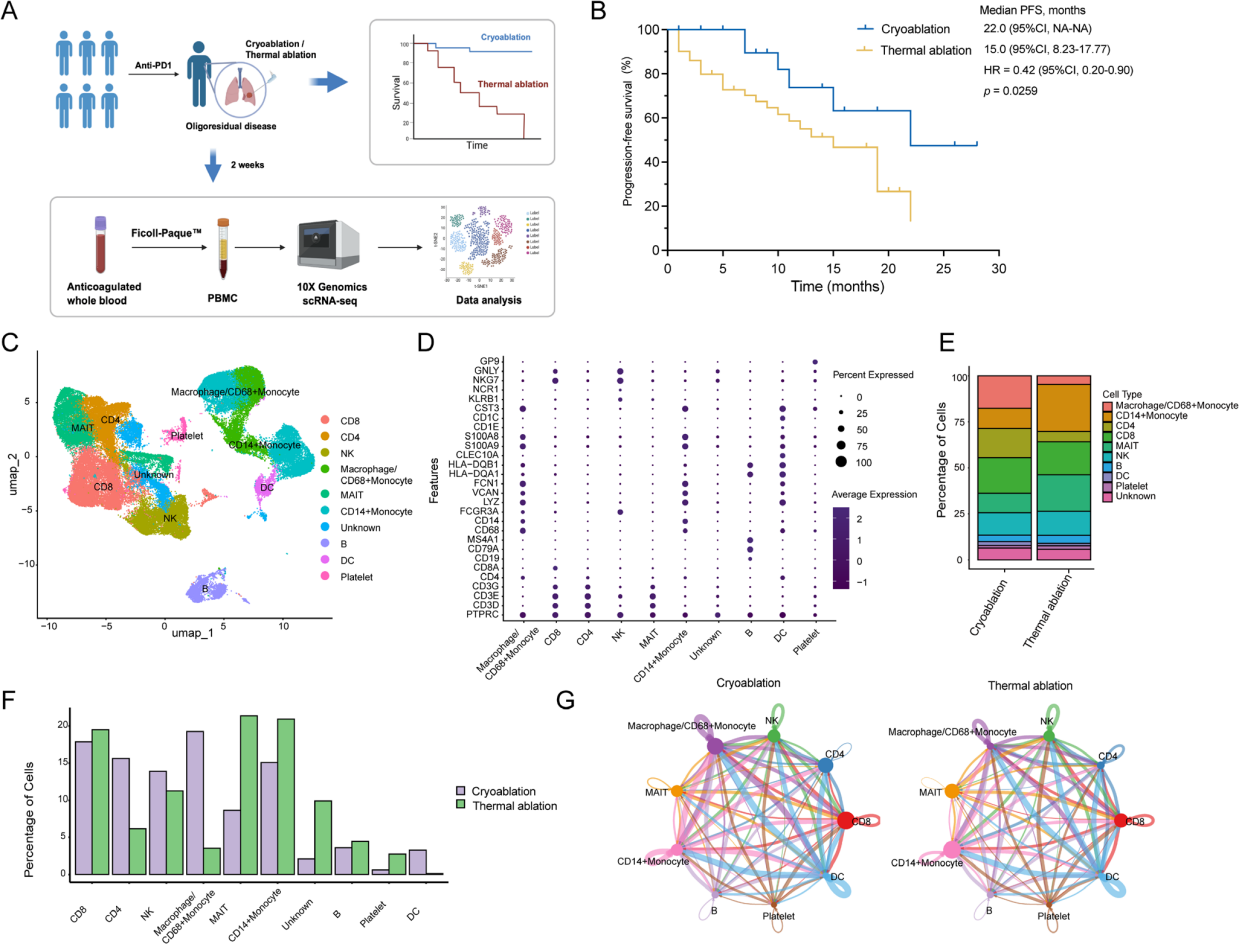
Survival analysis of cryoablation versus thermal ablation and immune microenvironment profiling of the PBMCs. (A) Schematic diagram of survival analysis and longitudinal peripheral‐blood sequencing in patients with oligometastatic disease who underwent either cryoablation or thermal ablation. (B) Kaplan–Meier estimates of PFS in NSCLC patients receiving immunotherapy combined with cryoablation (n = 26) or thermal ablation (n = 52). (C) UMAP visualization of PBMC scRNA‐seq from patients treated with cryoablation (n = 3) and thermal ablation (n = 3). Cells were classified into ten subpopulations based on distinct markers: CD8^+^ T cells, CD4^+^ T cells, NK cells, macrophages/CD68^+^ monocytes, MAIT cells, CD4^+^ monocytes, B cells, DCs, platelets, and unknown cells. (D) Bubble plot depicting the expression of canonical lineage markers defining of each cell population. (E) Stacked Bar chart showing relative abundances of immune‐cell subsets in cryoablation and thermal ablation groups. (F) Comparative bar chart of immune‐cell‐subset proportions between cryoablation and thermal ablation. (G) Cell–cell interaction networks of the predominant populations in the two ablation groups. The *p‐value* of B was determined by the log‐rank test (Mantel‐Cox). PFS, progression‐free survival.

To preliminarily assess whether cryoablation and thermal ablation confer differential efficacy in oligo‐residual lesions following immunotherapy, a total of 26 NSCLC patients who underwent cryoablation and 52 patients treated with microwave/radiofrequency ablation (MWA/RFA) were analyzed for survival (Figure 1A). The baseline characteristics of the patients are presented in Table S1. Compared with thermal ablation, cryoablation significantly prolonged progression‐free survival (PFS) (median PFS, 22.0 vs. 15.0 months; HR = 0.42, 95%CI:0.20‐0.97) (Figure 1B). To dissect the systemic immune alterations induced by cryoablation versus thermal ablation, we obtained PBMCs 2 weeks after ablation from three cryoablation‐treated patients and three MWA‐treated patients and subjected them to 10X Genomics scRNA‐seq. After stringent data preprocessing and quality control, a total of 65,609 immune cells were retained, comprising 33,959 cells from the cryoablation group and 31,650 cells from the thermal ablation group. After dimensionality reduction and graph‐based clustering, followed by canonical marker annotation, ten distinct immune subsets were clustered based on distinct markers (Figure 1C, D). Among them, the proportions of macrophages/CD68^+^ monocytes were higher after cryoablation than after MWA (Figure 1E, F). CellChat analysis further revealed that cryoablation markedly elevated the overall ligand–receptor interaction strength between macrophages/CD68^+^ monocytes and both CD8^+^ and CD4^+^ T cells compared with thermal ablation, with the CD4^+^ T cells showing a more pronounced enhancement (Figure 1G). These data indicated that macrophages, CD8^+^ T cells, and CD4^+^ T cells were likely the key cellular populations underlying the superior therapeutic efficacy of cryoablation over thermal ablation.

### 2.2 Macrophage Infiltration Increased in Mouse Tumors After Cryoablation

**FIGURE 2:**
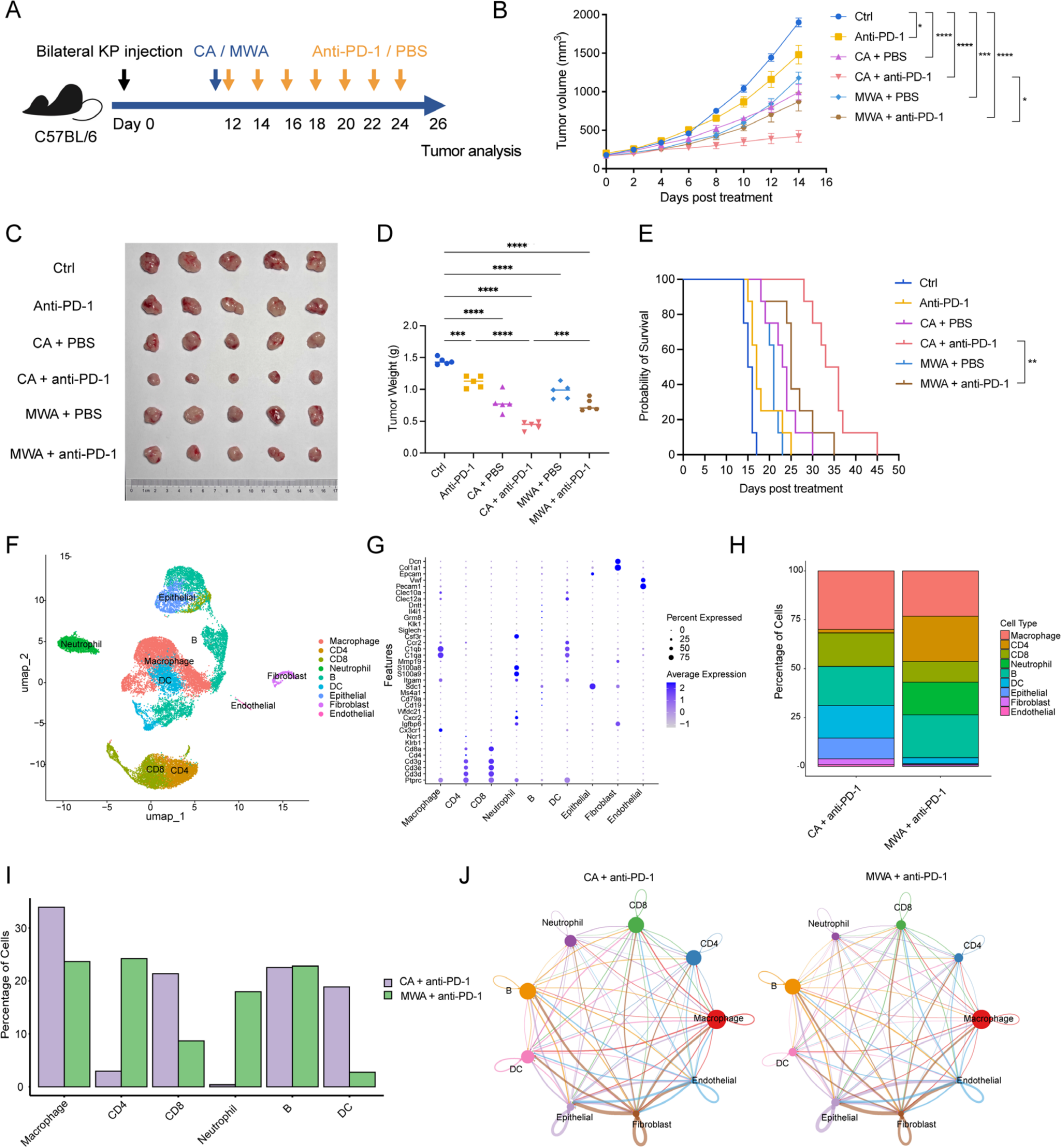
Therapeutic efficacy and immune microenvironment of cryoablation versus thermal ablation in mice. (A) Schematic diagram of ablation and anti‐PD‐1 treatment on mice. KP cells were injected subcutaneously into both flanks of C57BL/6 mice. when tumors reached 150–400 mm^3^, animals were randomized to six groups—control, anti‐PD‐1 monotherapy, cryoablation (CA) + PBS, microwave ablation (MWA) + PBS, MWA + anti‐PD‐1, and CA + anti‐PD‐1—and treated accordingly. (B) Tumor growth curves after therapy. C, D) Representative tumors (C) and tumor weights (D) 14 days post treatment. (E) Kaplan–Meier survival analysis of the six groups (n = 8 per group). (F) UMAP visualization of scRNA‐seq data from tumors harvested 14 d after MWA + anti‐PD‐1 and CA + anti‐PD‐1 therapy. (G) Representative marker gene expression for each cluster. (H) Stacked Bar Chart showing relative abundance of immune subpopulations in the two combination groups. (I) Comparative bar chart of immune‐cell‐subset proportions between CA + anti‐PD‐1 and MWA + anti‐PD‐1. (J) Cell–cell interaction networks among the major immune populations in the MWA + anti‐PD‐1 and CA + anti‐PD‐1 groups. B‐D, n = 5 mice/group; E, n = 8 mice/group. B and D were calculated using one‐way ANOVA. Tumor volumes and weights were shown as mean ± SEM. ^*^ *p* < 0.05, ^**^ *p* < 0.01, ^***^ *p* < 0.001, ^****^ *p* < 0.0001. SEM, standard error of the mean.

To substantiate the potentially superior antitumor activity of cryoablation over thermal ablation, *Kras^G12D/+^Tp53^−/−^* (KP) genetically engineered mouse model (GEMM)‐derived cells were inoculated into the bilateral flanks of C57BL/6 mice subcutaneously. When tumors reached 150–400 mm^3^, mice were randomly divided by volume into six groups: control, anti‐PD‐1 monotherapy, CA + PBS, microwave ablation (MWA) + PBS, MWA + anti‐PD‐1, and CA + anti‐PD‐1 (Figure 2A). Tumor‐growth curves monitored until day 14 post‐ablation revealed that single treatments modestly delayed progression, whereas combination regimens markedly suppressed contralateral (non‐ablated) tumors. Notably, the CA + anti‐PD‐1 group exhibited significantly smaller tumor volumes compared with the MWA + anti‐PD‐1 group (*p* < 0.05; Figure 2B, C), accompanied by reduced tumor weight (Figure 2D) and prolonged overall survival (Figure 2E). Neither significant changes in body weight nor histological alterations in major organs were observed following ablation (Figure S1A, B). Collectively, cryoablation achieved greater tumor suppression than thermal ablation without overtly affecting body weight. To uncover the immunological cellular mechanisms underlying the enhanced therapeutic efficacy of CA + anti‐PD‐1 in mouse models, we subjected non‐ablated tumors from the combination‐treatment group to scRNA‐seq and resolved nine distinct cell states based on canonical markers (Figure 2F, G). Compared with MWA, CA plus anti‐PD‐1 increased the proportional abundance of macrophages and CD8^+^ T cells within the distant tumor microenvironment (Figure 2H, I). Besides, in terms of both absolute interaction number and fold increase, CA plus anti‐PD‐1 yielded significantly higher communication strength between macrophages and CD8^+^ or CD4^+^ T cells than thermal ablation plus immunotherapy (Figure 2J). Collectively, these findings reaffirmed the critical role of macrophages in cryoablation combined with anti‐PD‐1 treatment.

### 2.3 Cryoablation Reactivated the Tumor Immune Microenvironment to Potentiate Immunotherapy

Previous analyses of human and mouse immune microenvironments identified macrophages as a key population mediating the antitumor efficacy of cryoablation combined with immunotherapy. To determine whether cryoablation can further improve therapeutic outcomes based on immunotherapy and how cryoablation reactivates the tumor immune microenvironment, we established NSCLC tumor models by subcutaneously inoculating KP, Lewis lung cancer (LLC), or *Kras* ^G12D/+^ *Lkb1* ^−/−^ (KL) cell lines into the bilateral flanks of C57BL/6 mice. When tumors reached 150–400 mm^3^, mice were randomized into four treatment groups: control, anti‐PD‐1 monotherapy, CA + PBS, or CA + anti‐PD‐1. Across all three models, combination therapy significantly retarded tumor growth, reduced tumor weight, and prolonged overall survival relative to either single agent (Figure S2A–I).

**FIGURE 3:**
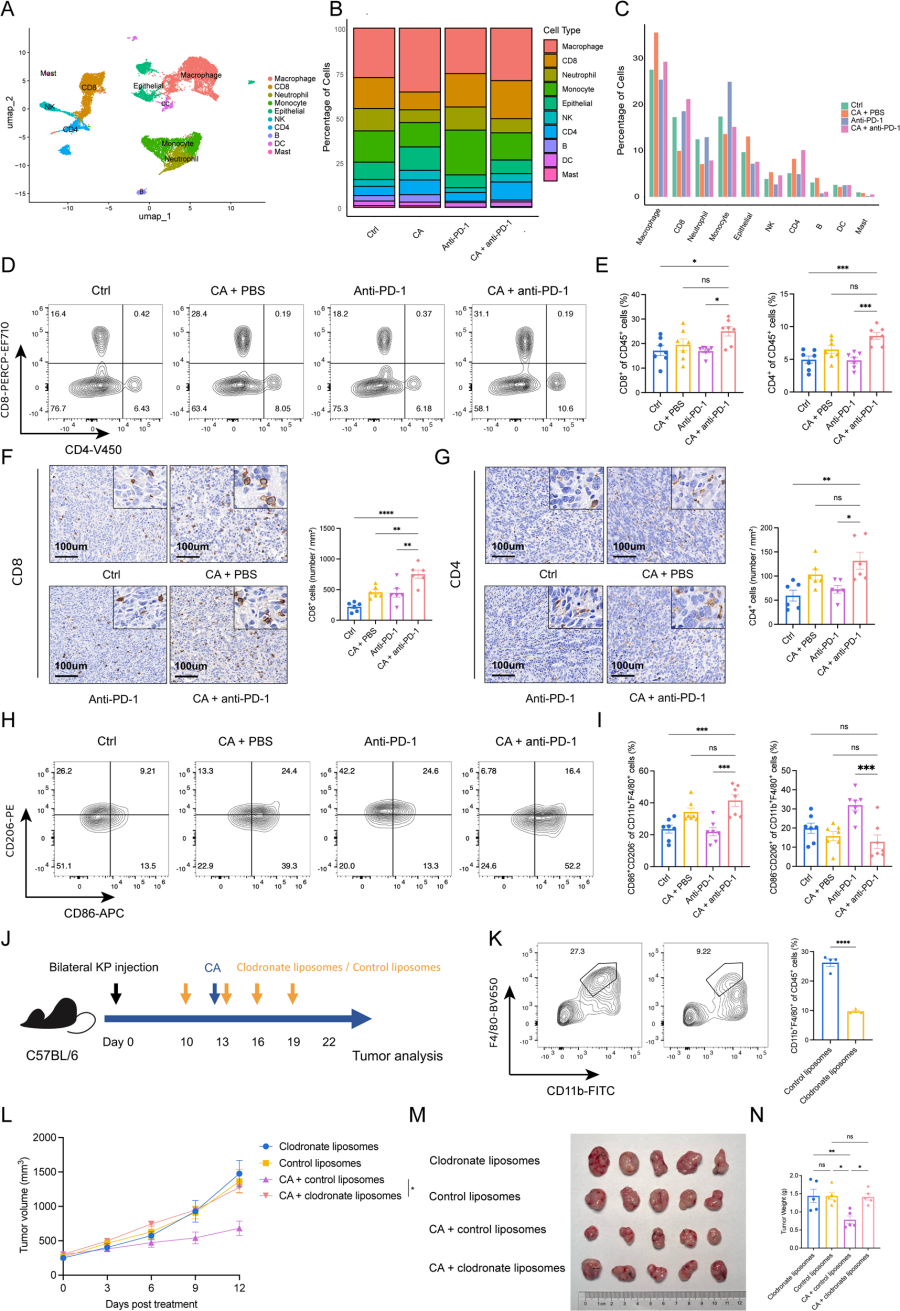
Cryoablation remodels macrophage and T cell landscapes in distant tumors. (A) In the KP tumor‐bearing mice receiving control, anti–PD‐1, CA + PBS, or CA + anti–PD‐1, tumors of the left side were harvested at day 14 after treatment for scRNA‐seq and displayed as the UMAP image. B, C) Stacked bar chart (B) and column graph (C) showing global cell‐type proportions across the four groups. D, E) Representative flow plots (D) and quantification (E) of intra‐tumoral CD8^+^ and CD4^+^ T cells. (F) Immunohistochemistry images (left) and density quantification (right) of CD8^+^ T cells. (G) Analogous IHC analysis for CD4^+^ T cells. H, I) Representative flow plots (H) and quantification of M1‐like (CD86^+^CD206^−^, left) and M2‐like (CD86^−^CD206^+^, right) macrophages (I). (J) Schematic diagram of macrophage depletion using clodronate‐liposome. (K) Representative flow cytometry plots (left) and quantification (right) of tumor tissues confirming macrophage depletion. (L) Tumor growth curves under macrophage‐depleted conditions. M, N) Photographs (M) and weights (N) of tumors 12 days after depletion. D and E, H and I, n = 7 mice/group; F and G, n = 6 mice/group; J–N) n = 5 mice/group. Data in E, F, G, I, and N were calculated using one‐way ANOVA. The *p‐values* of K and L were calculated using a two‐sided unpaired Student's *t‐*test. Data were shown as mean ± SEM. ns, not significant. ^*^ *p* < 0.05, ^**^ *p* < 0.01, ^***^ *p* < 0.001, ^****^ *p* < 0.0001. SEM, standard error of the mean.

To dissect the alterations in the tumor immune microenvironment, tumors from KP‐bearing mice were harvested 14 days after treatment, and CD45^+^ immune cells were isolated for scRNA‐seq. After quality control, 17,274 cells were subjected to dimensionality reduction and clustering (Figure 3A). Compared with anti‐PD‐1 alone, the CA + anti‐PD‐1 group exhibited a higher abundance of macrophages, CD8^+^ T cells, and CD4^+^ T cells (Figure 3B, C). We next used flow cytometry to further characterize the immune microenvironment, and the gating strategies are shown in Figure S3A, B. Flow cytometric analysis confirmed that adding cryoablation to PD‐1 blockade increased intra‐tumoral CD8^+^ and CD4^+^ T cells infiltration in KP and LLC models (Figure 3D, E). Functional profiling of T cells revealed expansion of CD69^+^CD8^+^, IFN‐γ^+^CD8^+^, and IFN‐γ^+^CD4^+^ populations, alongside heightened activation and enhanced cytotoxic capacity (Figure S4A–I). Immunohistochemistry of the KP model corroborated the elevated densities of CD8^+^ and CD4^+^ T cells within distal tumors of the combined group (Figure 3F, G), and similar results were observed in the LLC model (Figures S2J, K) and KL model (Figures S2L, M). Single‐cell analysis revealed that macrophages and T cells exhibited concordant trends between the monotherapy and combination groups (Figure 3C). Immunohistochemical analysis of KP tumor models revealed that combination therapy significantly promoted macrophage infiltration into tumor tissues compared with anti‐PD‐1 monotherapy (Figure S2N), and flow‐cytometric analysis was subsequently performed to characterize macrophages. Macrophages can be functionally divided into pro‐inflammatory M1‐like and anti‐inflammatory M2‐like subsets [21]. Compared with anti–PD‐1 monotherapy, the combination regimen significantly expanded the M1‐like (CD86^+^CD206^−^) compartment while reducing M2‐like (CD86^−^CD206^+^) macrophages (Figure 3H, I). Consistent with our observation that cryoablation increased macrophage abundance relative to thermal ablation in both patient PBMCs and mouse scRNA‐seq data, and that combination therapy further expanded macrophage numbers compared with anti‐PD‐1 alone, we hypothesized that macrophages are essential for the antitumor efficacy of cryoablation. To test this, we depleted macrophages using clodronate liposomes (Figure 3J, K, Figure S4J–L). Macrophage elimination abolished the therapeutic benefit of cryoablation, resulting in tumor weights significantly higher than those in the cryoablation group and indistinguishable from the control group (Figure 3L–N). Macrophages and T cells engage in critical reciprocal interactions, and both human PBMCs and mouse tumors exhibited elevated T‐cell infiltration after cryoablation. To determine whether T cells are indispensable for cryoablation efficacy, we depleted CD8^+^ or CD4^+^ or T cells in tumor‐bearing mice (Figure S5A). Flow‐cytometry confirmed near‐complete elimination of peripheral CD8^+^ and CD4^+^ T cells following depletion (Figure S5B–G). Following depletion of CD4^+^ and CD8^+^ T cells, the antitumor efficacy of cryoablation combined with anti‐PD‐1 therapy was abolished, with both tumor volume and weight significantly increased compared with the IgG control group (Figure S5H, I, K, L). Survival analysis revealed that loss of either subset abolished the prolonged anti‐tumor benefit conferred by cryoablation (Figure S5J, M), indicating that cryoablation‐induced tumor suppression was mediated through macrophage‐regulated activation of T cells.

To assess long‐term immune maintenance, we analyzed splenic central memory T cells (Tcm) and observed significantly increased CD4^+^ and CD8^+^ Tcm populations in the combination group (Figure S6A–C). We then established a tumor rechallenge model (Figure S6D), which revealed significantly reduced tumor volume and weight in the rechallenge group compared with controls (Figure S6E–G). These findings indicated that cryoablation combined with anti‐PD‐1 induced durable anti‐tumor immune memory in NSCLC.

### 2.4 CXCL10^+^ Macrophages Displayed a Critical Antitumor Role in Cryoablation‐Induced Immunity

**FIGURE 4:**
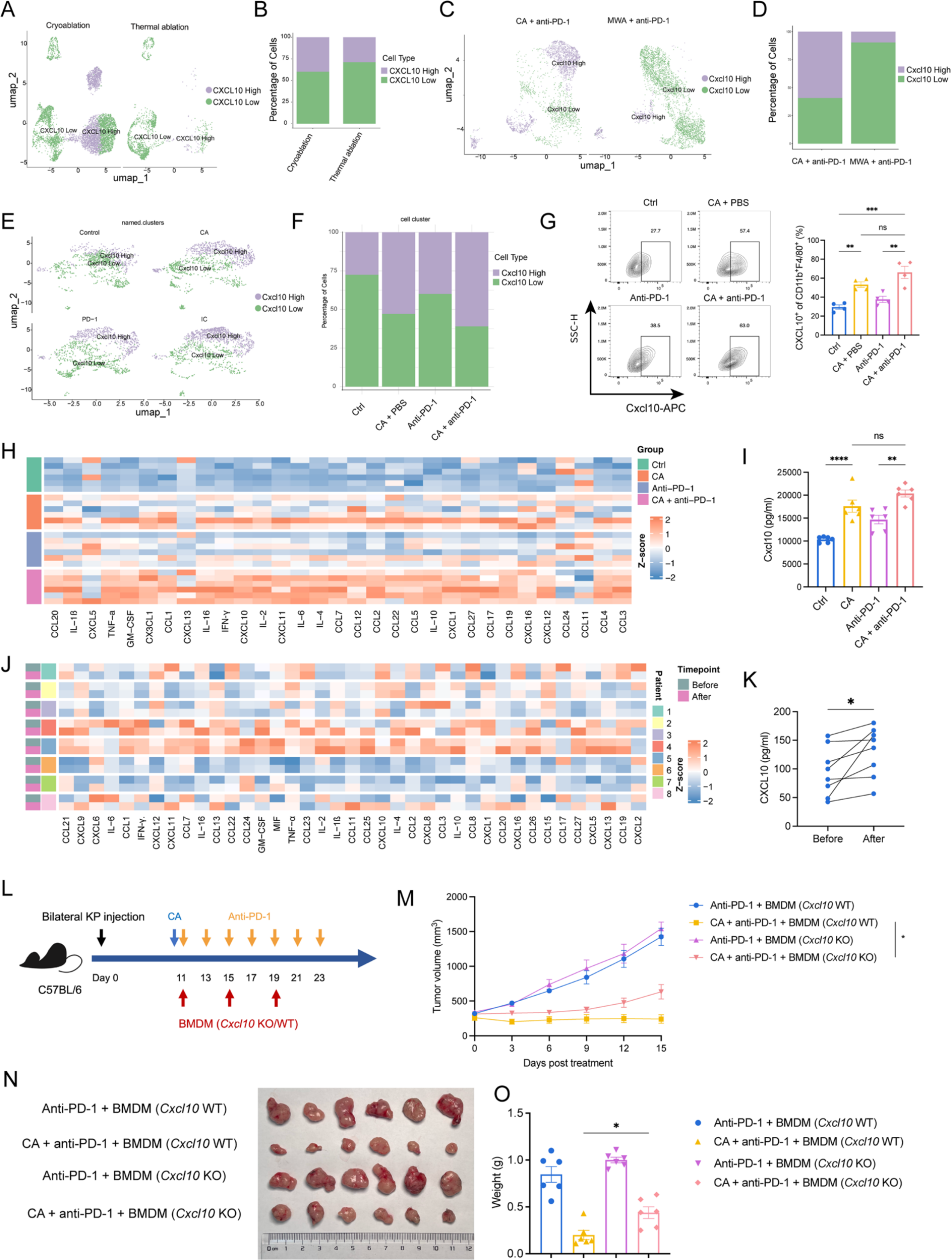
Cryoablation enriches CXCL10^+^ macrophages in the immune microenvironment. A, B) UMAP projection (A) and quantification (B) of CXCL10‐high and CXCL10‐low macrophages in patient PBMCs. C, D) UMAP plots (C) and proportional bar graph (D) of *C* *xcl10*‐high vs. *Cxcl10* ‐low macrophages in KP tumors after cryoablation + anti–PD‐1 or microwave ablation (MWA) + anti–PD‐1. E, F) UMAP visualization (E) and cell fractions (F) of CXCL10‐high and CXCL10‐low macrophages across control, anti–PD‐1 alone, CA + PBS, and CA + anti–PD‐1 groups. (G) Representative flow cytometry plots (left) and quantification (right) of intra‐tumoral CXCL10^+^ macrophages. H, I) Heat maps of serum multiplex cytokine profiles from mice (H) and quantified Cxcl10 levels across the four groups (I). J, K) Heat maps of serum multiplex cytokine profiles before and after cryoablation (J) and corresponding CXCL10 quantification (K) in patients. (L) Schematic diagram of the BMDM adoptive transfer experiment. C57BL/6 mice received tail vein injection of 1 × 10^5^ *C* *xcl10*‐KO or *Cxcl10*‐WT BMDMs on days 0, 3, and 6 post treatment. M–O) Tumor growth curves (M), tumor tissues at day 15 post‐treatment (N), and tumor weights (O). G, n = 4 mice/group; H and I, n=6 mice/group; J and K, n=8 patients; L‐O, n = 4 mice/group. G, I, M, and O were calculated using one‐way ANOVA. The *p‐*value of K was calculated using a two‐sided paired Student's *t‐*test. Data were shown as mean ± SEM. Data were shown as mean ± SEM. ns, not significant. ^*^ *p* < 0.05, ^**^ *p* < 0.01, ^***^ *p* < 0.001, ^****^ *p* < 0.0001. SEM, standard error of the mean.

To pinpoint which subset of macrophages was responsible for the antitumor efficacy of cryoablation, we stratified macrophages into CXCL10‐high and CXCL10‐low expression subpopulations using CXCL10 as the discriminative marker. Analysis of human PBMCs and mouse tumor scRNA‐seq datasets revealed a higher infiltration of CXCL10^+^ macrophages in the cryoablation plus anti‐PD‐1 group than in the thermal‐ablation combined with anti‐PD‐1 group (Figure 4A–D). Furthermore, based on the macrophage‐related gene set, dimensionality reduction and clustering analysis revealed that cryoablation promotes an increase in M1‐like macrophages (Figure S7B, C), and this cell population highly overlapped with CXCL10^+^ macrophages, suggesting that CXCL10^+^ macrophages exhibit a pro‐inflammatory phenotype. Similarly, cryoablation combined with anti‐PD‐1 yielded more CXCL10^+^ macrophages than anti‐PD‐1 monotherapy, a trend corroborated by flow cytometry (Figure 4E–G). The proportion of CXCL10^+^ macrophages was up‐regulated in both the cryoablation versus control comparison and the combination therapy versus anti‐PD‐1 treatment comparison (Figure 4G). As a previous study has demonstrated, CXCL10 secreted by macrophages into the peripheral circulation could exert chemotactic effects [22]. We therefore quantified CXCL10 levels in the peripheral blood of both mice and patients to determine the change of CXCL10 after cryoablation. Multiplex cytokine profiling of serum collected 14 days after treatment in KP cells bearing mice showed marked elevations of IFN‐γ, TNF‐α, and CXCL10 in the combination cohort (Figure 4H, I). Consistently, paired sera of patients demonstrated a significant post‐ablation increase in circulating CXCL10 (Figure 4J, K). Collectively, these data indicated that cryoablation augmented systemic CXCL10^+^ macrophage abundance in both the peripheral blood and distant tumors, while simultaneously enhancing serum CXCL10 secretion. To further investigate whether CXCL10^+^ macrophages play a critical role in combination therapy, *Cxcl10*‐KO and *Cxcl10*‐WT BMDMs were adoptively transferred via tail vein injection into tumor‐bearing mice (Figure 4L). Our results demonstrated that adoptive transfer of *Cxcl10*‐WT BMDMs further enhanced the antitumor efficacy of combination therapy, as evidenced by markedly suppressed tumor growth and significantly reduced tumor volume (Figure 4M–O). Collectively, these data indicated that CXCL10^+^ macrophages supported cryoablation and anti‐PD‐1‐mediated antitumor effects.

### 2.5 Cryoablation Expanded the CXCR3^+^ T Cells to Exert a Critical Antitumor Effect

**FIGURE 5:**
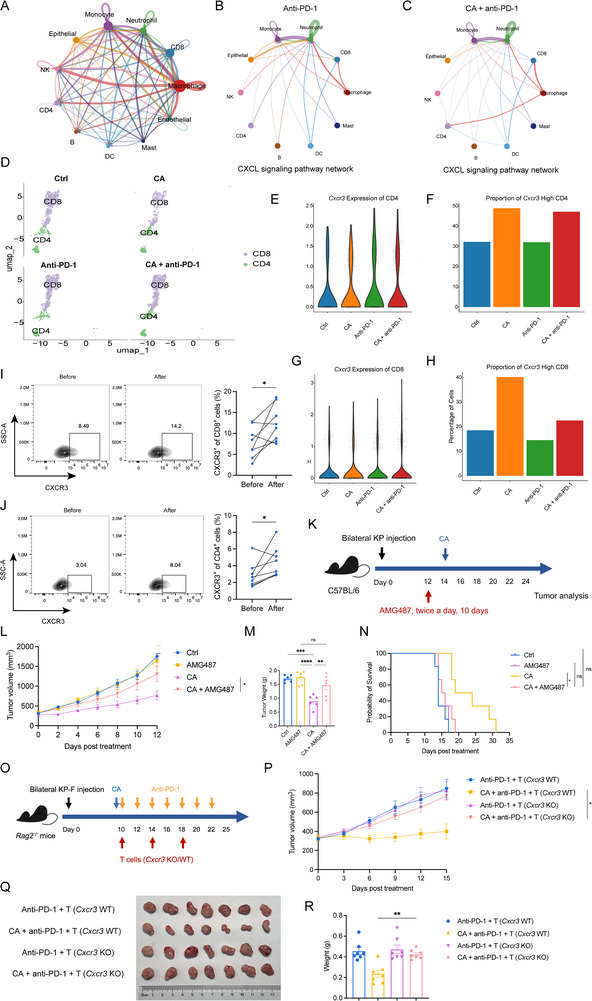
Cryoablation drives CXCR3^+^ T cell recruitment into the tumor microenvironment. (A) Global cell–cell communication networks among control, anti–PD‐1, CA + PBS, and CA + anti–PD‐1 groups. B, C) CXCL‐signaling communication maps for anti‐PD‐1 monotherapy (left) and the combination therapy (right). (D) UMAP of CD8^+^ and CD4^+^ T cells across the four treatment groups. E, F) Violin (E) and bar (F) plots quantifying CXCR3^+^CD4^+^ T‐cell abundance. G, H) Violin (G) and bar (H) plots quantifying CXCR3^+^CD8^+^ T‐cell abundance. I) Representative flow plots (left) andx quantification (right) of CXCR3^+^CD8^+^ T cells in patient PBMCs. (J) Representative flow plots (left) and quantification (right) of CXCR3^+^CD4^+^ T cells in patient PBMCs. (K) Experimental scheme of CXCR3^+^ T cells blockade with AMG487. AMG487 was administered twice a day subcutaneously for 10 consecutive days. L–N) tumor growth curves (L), excised tumor weights (M), and Kaplan–Meier survival curves (N) for the CXCR3‐blockade experiment. O, Schematic diagram of the T cells adoptive transfer experiment. Rag2^−^/^−^ mice received tail vein injection of 1 × 10^5^ sorted splenic T cells from *C* *xcr3*‐KO or *Cxcr3*‐WT donors on days 0, 3, and 6 post treatment. P–R) Tumor growth curves (P), tumor tissues at day 15 post‐treatment (Q), and tumor weights (R). I and J, n=8 samples / group; K‐N, n=6 mice / group; O‐R, n=7 mice / group. The *p‐*values of I and J were calculated using a two‐sided paired Student's *t‐*test. The *p‐*values of M, P, and R were calculated using one‐way ANOVA. Data were shown as mean ± SEM. *P* values of N were determined by the log‐rank test (Mantel‐Cox). ns, not significant. ^*^ *p* < 0.05, ^**^ *p* < 0.01, ^***^ *p* < 0.001. SEM, standard error of the mean.

The receptor for CXCL10 on macrophages is CXCR3, which is mainly expressed on T cells [23]. We therefore hypothesized that CXCL10^+^ macrophages could recruit additional CXCR3^+^ T cells into the tumor microenvironment to exert a potent antitumor effect. Cell–cell communication analysis of the four groups (Ctrl, anti‐PD‐1, CA + PBS, and CA + anti‐PD‐1) revealed robust interactions between macrophages and both CD8^+^ and CD4^+^ T cells (Figure 5A). Detailed interrogation showed that the CXCL signaling pathway was markedly stronger in the combination group than in the anti‐PD‐1 monotherapy group (Figure 5B, C). Sub‐clustering of T cells across the four groups (Figure 5D) demonstrated a significant increase in the proportion of CXCR3^+^ cells within both CD8^+^ and CD4^+^ T cells after the combination treatment (Figure 5E–H). Flow cytometric analysis of peripheral blood of patients collected two weeks post‐ablation confirmed a pronounced expansion of CXCR3^+^ CD8^+^ T cell and CXCR3^+^ CD4^+^ T cell subsets (Figure 5I, J), as well as IFN‐γ^+^ CD4^+^ and IFN‐γ^+^ CD8^+^ T‐cell subpopulations (Figure S7D–F). To functionally validate the contribution of CXCR3^+^ T cells, we pharmacologically disrupted the CXCL10–CXCR3 axis using AMG487 (Figure 5K). Blocking the recruitment of CXCR3^+^ T cells by CXCL10 completely abolished the antitumor efficacy of cryoablation, resulting in accelerated tumor growth, increased tumor weight, and shortened survival of mice than the single cryoablation group (Figure 5L–N). To further validate the critical role of CXCR3^+^ T cells in mediating the anti‐tumor efficacy of combined cryoablation and anti‐PD‐1 therapy, splenic T cells from *Cxcr3*‐WT and *Cxcr3*‐KO mice were sorted and adoptively transferred into *Rag* *2^−/−^* recipients via tail vein injection (Figure 5O, Figure S7G, H). Our results demonstrated that adoptive transfer of *Cxcr3*‐WT T cells, but not *C* *xcr3* ‐KO T cells, significantly enhanced the anti‐tumor efficacy of CA + anti‐PD‐1 therapy, as evidenced by suppressed tumor growth and reduced tumor weight (Figure 5P–R). Overall, these data supported that the CXCR3^+^ T cells recruited by CXCL10‐producing macrophages after cryoablation constituted the functionally active population driving antitumor immunity.

### 2.6 Intra‐Tumoral CXCR3^+^ T Cells Expanded by Cryoablation Were Recruited From the Draining Lymph Nodes

**FIGURE 6:**
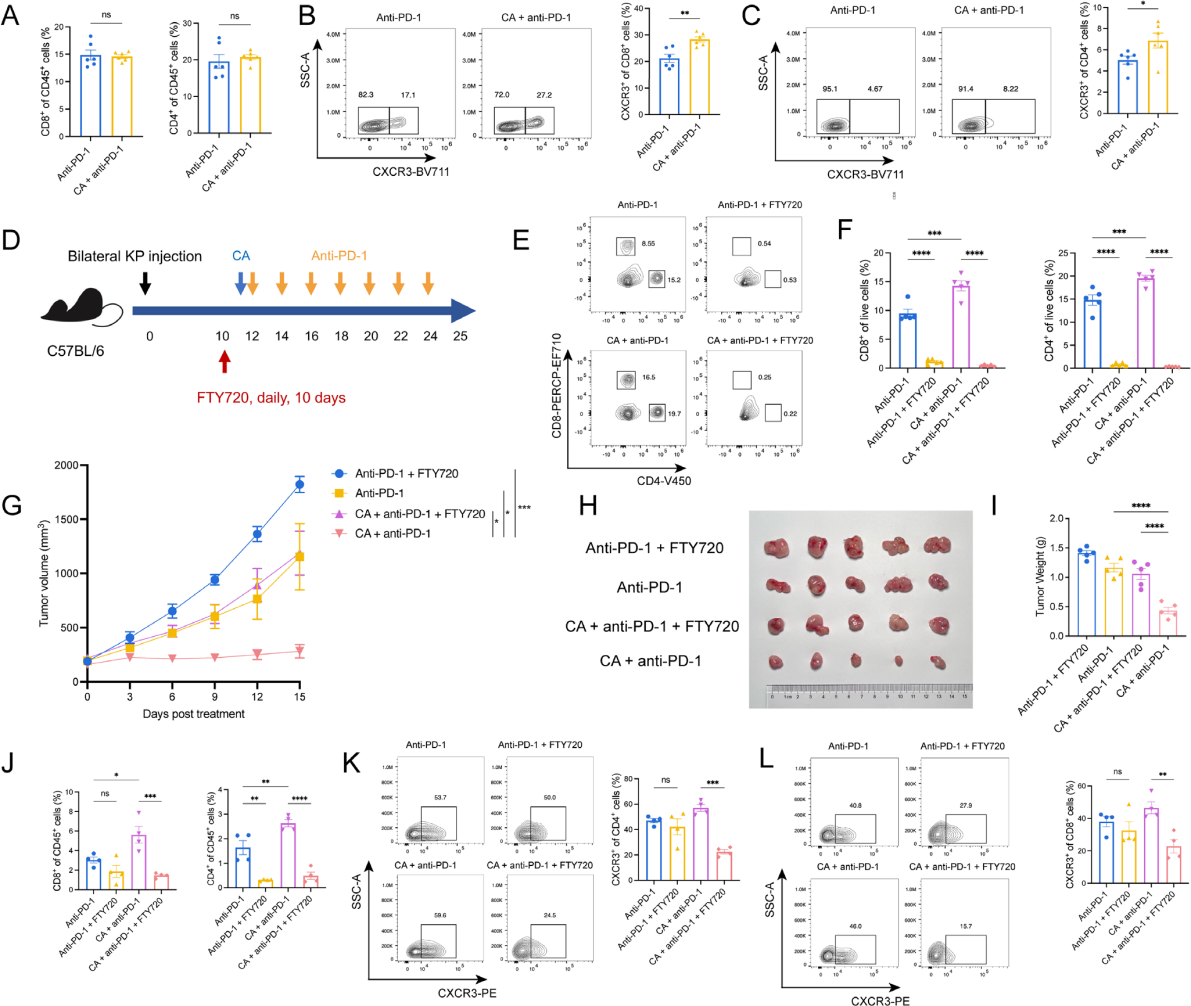
FTY720 abrogated the antitumor efficacy of cryoablation. (A) CD8^+^ (left) and CD8^+^ (right) T cells in draining lymph nodes after anti–PD‐1 or CA + anti–PD‐1 treatment. (B) Representative flow plots (left) and quantification (right) of CXCR3^+^CD8^+^ T cells in lymph nodes from the same groups. (C) Analogous analysis for CXCR3^+^CD4^+^ T cells. (D) An experimental schema of mice received daily oral FTY720 for 10 consecutive days to block T cells' egress from lymph nodes. E, F) Flow cytometry confirmation of peripheral depletion of T cells: representative PBMC plots (E) and summary data for CD8^+^ (F left) and CD4^+^ (F right) T cells after FTY720 treatment. G–I) Tumor growth curves (G), photographs (H), and excised tumor weights (I) for the four treatment arms under FTY720 blockade (n = 5 per group). (J) Intra‐tumoral CD8^+^ (left) and CD4^+^ (right) T‐cell proportions determined by flow cytometry. K–L, CXCR3^+^CD4^+^ (K) and CXCR3^+^CD8^+^ (L) T‐cell infiltration in the lymph node blockade experiment: representative flow cytometry plots (left) and quantification (right). A–C) n = 6 mice/group; n = 4 per group for J‐L. The *p‐*values of A–C were calculated using a two‐sided unpaired Student's *t‐*test. The *p‐*values of (F) I–L) were calculated using one‐way ANOVA. Data were shown as mean ± SEM. ns, not significant. ^*^ *p* < 0.05, ^**^ *p* < 0.01, ^***^ *p* < 0.001, ^****^ *p* < 0.0001. SEM, standard error of the mean.

Draining lymph nodes (dLNs) were the principal sites for peripheral T‐cell priming, expansion, and functional differentiation [24]. We therefore performed flow cytometry on inguinal dLNs from mice receiving anti‐PD‐1 alone and combined with cryoablation. Although total CD8^+^ and CD4^+^ T cells in dLNs were comparable between the two groups, the fractions of CXCR3^+^CD8^+^ T and CXCR3^+^CD4^+^ cells were significantly elevated after combination treatment (Figure 6A–C). To determine whether the increased intra‐tumoral CXCR3^+^ T cells originated from dLNs, we blocked T‐cell egress from lymph nodes with FTY720 treatment (Figure 6D). Peripheral‐blood analysis confirmed near‐complete absence of circulating CD8^+^ and CD4^+^ T cells (Figure 6E, F). Consequently, the tumor‐growth control mediated by cryoablation plus anti‐PD‐1 was completely abolished, resulting in markedly larger tumors and higher tumor weights (Figure 6G–I). Intratumor flow cytometry further revealed a profound reduction in CXCR3^+^CD8^+^ and CXCR3^+^CD4^+^ T cells (Figure 6J–L). To exclude the potential contribution of NK cells to the anti‐tumor efficacy of cryoablation combined with anti‐PD‐1 therapy, we performed NK cell depletion in mice, validated depletion efficiency by flow cytometric analysis of splenocytes, and monitored tumor growth curves as well as tumor weight at the end of treatment (Figure S8D–F). As shown in Figure S8G–I, NK cell depletion was found to have a limited impact on therapeutic efficacy. These data demonstrated that cryoablation could expand CXCR3^+^ T cells in dLNs, which then egressed and were recruited into distant tumors via the CXCL10‐CXCR3 axis to mediate antitumor immunity.

### 2.7 Cryoablation Activated the cGAS–STING Signaling in Macrophages to Potentiate Immunotherapy

**FIGURE 7:**
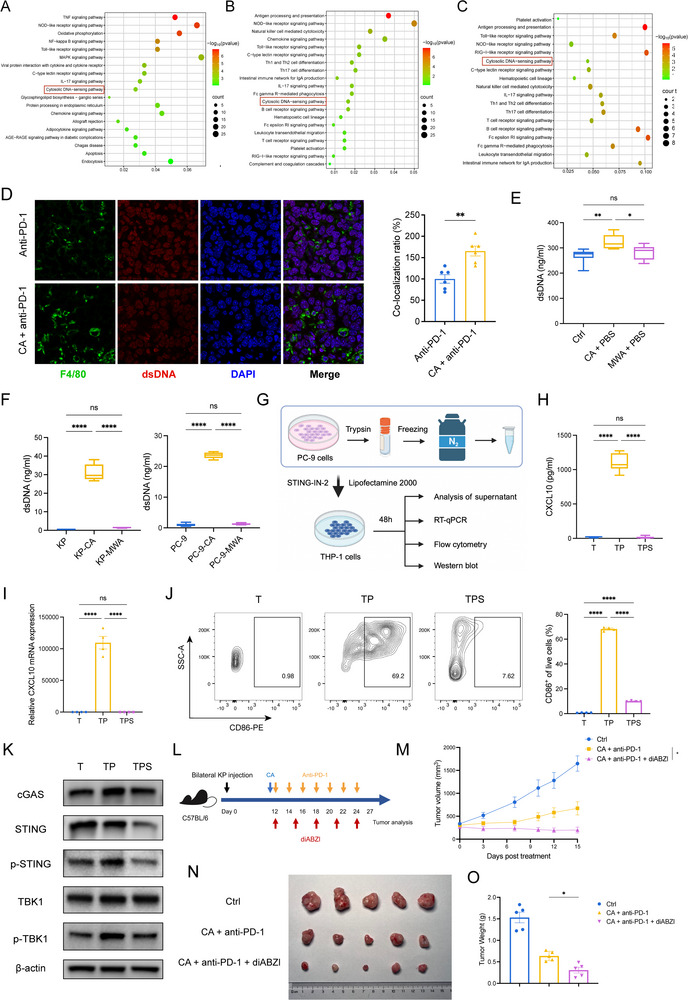
Cryoablation activates the cGAS–STING signaling in macrophages. (A) Bubble plots of enriched pathways in PBMC macrophages from patients treated with cryoablation versus thermal ablation. (B) Pathway enrichment bubble plots comparing tumor macrophages from CA + anti‐PD‐1 versus MWA + anti‐PD‐1 treated mice. (C) Pathway enrichment analysis for CA + anti‐PD‐1 compared to anti‐PD‐1 alone. (D) Immunofluorescence staining (left) and quantitative analysis of fluorescence colocalization (right) of distal tumors from the anti‐PD‐1 and CA + anti‐PD‐1 groups after ablation. (E) Serum dsDNA quantification in mice from Ctrl, CA+PBS, and MWA+PBS groups at 7 days post‐ablation. (F) dsDNA quantification in cell supernatants from KP and PC‐9 cells after cryoablation and microwave ablation, respectively. Liquid nitrogen was used to simulate cryoablation, and a water bath was used to simulate microwave ablation. (G) In vitro stimulation schema: Resuspend PC‐9 cells in PBS, lyse in liquid nitrogen, and centrifuge the supernatant, which was complexed with Lipofectamine 2000 and transfected into THP‐1 cells for culture of 48 h with or without Sting‐IN‐2. H, I) ELISA of secreted CXCL10 in culture supernatants (H) and qPCR for CXCL10 mRNA (I). (J) Flow‐cytometry plots (left) and quantification (right) of CD86^+^ THP‐1 cells. (K) Western blot of cGAS–STING pathway proteins 48 h post stimulation. (L) Experimental design of STING agonist treatment based on CA + anti‐PD‐1. M–O) Tumor growth curves (M), representative tumors (N), and excised tumor weights (O) under combined treatment; n=5 per group. T, THP‐1 cells; TP, THP‐1 cells incubated with supernatant of PC‐9 tumor cells lysed in liquid nitrogen; TPS, THP‐1 cells incubated with supernatant of PC‐9 tumor cells lysed in liquid nitrogen and STING‐IN‐2. The *p‐*values of F, H J, M, and O were calculated using one‐way ANOVA. Data were shown as mean ± SEM. ns, not significant. ^*^ *p* < 0.05, ^****^ *p* < 0.0001. SEM, standard error of the mean.

To elucidate how cryoablation activates macrophages to exert antitumor effects, we performed differential gene‐expression and pathway‐enrichment analyses on macrophages purified from patient PBMCs after either thermal or cryoablation. Compared with thermal ablation, cryoablation significantly up‐regulated the cytosolic DNA‐sensing pathway in macrophages (Figure 7A). Consistently, CXCL10‐high versus CXCL10‐low macrophage subsets from mouse tumors, as well as macrophages from the combination‐therapy versus anti‐PD‐1 monotherapy group, also displayed enrichment of the cytosolic DNA‐sensing pathway (Figure 7B, C). The cytosolic DNA‐sensing pathway can activate the downstream cGAS‐STING signaling pathway. Previous studies have reported that the cGAS‐STING pathway is also essential for DC activation. To explore which cell type plays a more critical role, we scored the gene sets of the cGAS‐STING pathway in DCs and macrophages. As shown in Figure S9A, B, cryoablation activated the cGAS‐STING pathway in DCs. However, given the baseline differences and dynamic changes in macrophage populations. We therefore hypothesized that tumour‐derived DNA released by cryoablation was taken up by macrophages, triggering cGAS–STING signaling, driving CXCL9/10 secretion and M1 polarization, and ultimately recruiting CXCR3^+^ T cells. To test this, we performed immunofluorescence staining for macrophages and dsDNA in non‐ablated tumors from mice receiving immunotherapy and cryoablation combined with immunotherapy. Tumors in the cryoablation combined with immunotherapy group contained more macrophages and engulfed more dsDNA relative to the immunotherapy group (Figure 7D). In addition, serum analysis from mice at day 7 post‐ablation revealed significantly elevated dsDNA levels following cryoablation compared to both the control and MWA groups (Figure 7E). To further elucidate this phenomenon mechanistically, we performed H&E staining on ablated tumor tissues collected 48 h after cryoablation or MWA. Cryoablation induced disruption of both cell membranes and nuclei, whereas tumor tissues after MWA exhibited relatively preserved cellular morphology (Figure S9C). Further analysis of cell death modes induced by both ablation methods showed that cryoablation primarily induced apoptosis and pyroptosis, whereas thermal ablation mainly caused necrosis (Figure S9D). We further simulated cryoablation and thermal ablation in vitro using liquid nitrogen and water bath treatment, respectively, on KP and PC‐9 cells. Centrifuged cell supernatants similarly demonstrated higher dsDNA concentrations following liquid nitrogen lysis (Figure 7F). To further validate dsDNA engulfment by macrophages, the centrifuged supernatant from liquid nitrogen‐treated PC‐9 cells was added to THP‐1 macrophages in vitro using Lipofectamine 2000. (Figure 7G). ELISA of the culture supernatant 48h after transfection showed that KP lysate markedly increased the CXCL10 secretion of THP‐1, whereas co‐incubation with the STING inhibitor STING‐IN‐2 abolished this effect (Figure 7H). RT‐qPCR confirmed a corresponding change in *Cxcl10* mRNA levels (Figure 7I). Flow cytometry revealed a pronounced expansion of M1 (CD86^+^) macrophages, an effect completely reversed by STING‐IN‐2 (Figure 7J). Western blot further showed enhanced phosphorylation of STING and TBK1 in the RK group, and addition of the STING inhibitor largely abrogated these phosphorylation signals (Figure 7K). We stimulated RAW264.7 and BMDMs with KP cells using the same approach, yielding comparable results (Figure S9E–H, J, K). Additionally, non‐ablated tumor tissues from mice revealed enhanced activation of the cGAS‐STING pathway in the combination therapy group compared to immunotherapy alone (Figure S9I). These assays demonstrated that tumor‐cell lysate supernatant could activate the cGAS–STING signaling pathway in macrophages and promote CXCL10 cytokine secretion. To validate the results in vivo, the STING agonist diABZI [25], which induces a more dramatic activation of STING signaling, was added to the CA + anti‐PD‐1 treatment. The triple‐combination therapy further amplified tumor growth inhibition in KP‐bearing mice, producing the most pronounced survival benefit observed in this study (Figure 7J–M). These in vivo and in vitro experiments revealed that cryoablation‐triggered cGAS–STING activation in macrophages was necessary to potentiate the therapeutic efficacy of anti‐PD‐1 treatment.

## 3 Discussion

Tumor ablation is an important component of modern cancer therapy, with the two most widely used modalities being thermal ablation and cryoablation. [26, 27]. In this study, we first found that cryoablation produced significantly better clinical outcomes than thermal ablation when combining with PD‐1/PD‐L1 inhibitors in patients with advanced NSCLC, as well as preclinical animal models. By constructing a comprehensive single‐cell transcriptomic atlas from clinical PBMC samples, whole tumor lesions in mice, and CD45^+^ immune cells, we observed a significantly higher abundance of macrophages in both the PBMCs of cryoablation‐treated patients and the non‐ablated tumor lesions of cryoablation‐treated mice compared with their thermal‐ablation counterparts. Furthermore, these macrophages exhibited high expression of CXCL10. Furthermore, we observed that the plentiful tumor‐derived dsDNA released is predominantly taken up by macrophages, specifically activating the cGAS‐STING signaling and driving their differentiation into CXCL10+ macrophages after the cryoablation. Thereafter, these CXCL10+ macrophages migrate to distant lesions and, via the CXCL10‐CXCR3 chemotactic axis, effectively recruit T lymphocytes, thereby enhancing the efficacy of PD‐1 inhibitor immunotherapy.

More and more evidence has shown that cryoablation offers clear advantages in the treatment of various tumors [26, 27, 28, 29]. However, the immune response induced by cryoablation alone may be insufficient to generate durable systemic antitumor effects [5]. Therefore, combination strategies such as adoptive cell transfer or immunotherapy were investigated [19, 30, 31, 32, 33]. As we know, ICIs have dramatically changed the landscape of anti‐cancer therapy [34, 35] and have become a standard treatment [34, 36, 37] for patients with locally advanced or lung cancer. Our study showed that combining ICIs with cryoablation produced stronger synergistic antitumor effects than ICIs alone, cryoablation alone, or thermal ablation combined with ICIs in the animal model. More importantly, we first observed that cryoablation combined with immunotherapy provided superior outcomes than thermal ablation in patients with lung cancer, which would be helpful to guide the choice of ablation when combined with immunotherapy.

Previous studies had shown that tumor‐infiltrating CD11b^+^ dendritic cells (CD11b^+^ DCs) could sense tumor‐derived dsDNA, activate the cGAS–STING pathway, and induce type I interferon production, which played a critical role in antitumor immunity [38]. However, it is still unknown why cryoablation can more effectively reshape immune‐cell composition and function than thermal ablation. Our study found that compared with thermal ablation, cryoablation induces significantly more immunogenic cell death processes, such as apoptosis and pyroptosis, during which substantial amounts of tumor‐derived dsDNA are released, which activated the cGAS–STING signaling in peripheral monocytes/macrophages and drove their differentiation toward a CXCL10‐high macrophage phenotype. Although our data indicate that cryoablation can also activate the cGAS–STING signaling pathway in DCs, given the greater abundance and phenotypic differences of macrophages, we propose that macrophages likely play a more central role in this process. CXCL10^+^ macrophages not only act locally at the primary lesion but can also migrate to distant lesions, efficiently recruit and concentrate CXCR3‐expressing effector T cells. Moreover, the polarization of CXCL10+ macrophages toward an M1‐like phenotype following cGAS‐STING activation is critical for the success of anti‐tumor immune responses. Its direct downstream effects include robust production of type I interferons and interferon‐induced chemokines (such as CXCL10), which not only enhance their own anti‐tumor efficacy but also promote the infiltration and cytotoxic function of CXCR3+ T cells at distant lesion sites. Ultimately, this process enhances the effectiveness of PD‐1 inhibitors and other immunotherapies.

Theoretically, cryoablation induces immunogenic cell death (ICD) through repeated freeze–thaw cycles [26]. During slow thawing, intracellular ice crystals rupture the plasma and nuclear membranes, releasing large amounts of intact genomic dsDNA into the extracellular space [26]. ScRNA‐seq and immunological evidence indicate that compared with thermal ablation, cryoablation induces more extensive apoptosis and pyroptosis in cells, thereby releasing a greater amount of double‐stranded DNA. This DNA is taken up by tumor‐resident macrophages, triggering their transcriptional reprogramming. This reprogramming is characterized by upregulation of CXCL10 and type I interferon‐related genes, leading to the formation of a CXCL10^+^ macrophage cluster with immunostimulatory functions. The intra‐tumoral macrophage is largely replenished by strong recruitment of peripheral monocytes driven by tumor‐derived factors such as CCL‐2 and CSF‐1 [39], and cryoablation can promote activation of CXCL10^+ ^macrophages in peripheral blood and their subsequent recruitment into the tumor, where they exert tumor‐suppressive functions. As a result, the dsDNA–cGAS–STING–type I IFN and related systemic signals triggered at the primary site by cryoablation pre‐activate circulating monocytes into a CXCL10^+^ phenotype, create chemical and physical “passcodes” that recruit and allow engraftment of these circulating CXCL10^+^ macrophages.

This study has several limitations. First, the superior efficacy of cryoablation with immunotherapy was from our retrospective analysis and lacked the evidence from prospective randomized trials. Second, further dissection of the relative functional contribution of CXCL10^+^ macrophages compared with other immune cells in augmenting immunotherapy after cryoablation might provide more meaningful information. Thirdly, only partial features of CXCL10^+^ macrophages and the precise mechanisms regulating their migration were explored in this study. Therefore, approaches such as cell‐tracking, receptor/ligand blockade, and spatial‐omics are needed to define the origin of these cells, their migration receptors and routes, to enable more precise clinical modulation of this process and synergy with immunotherapy in the future.

**FIGURE 8:**
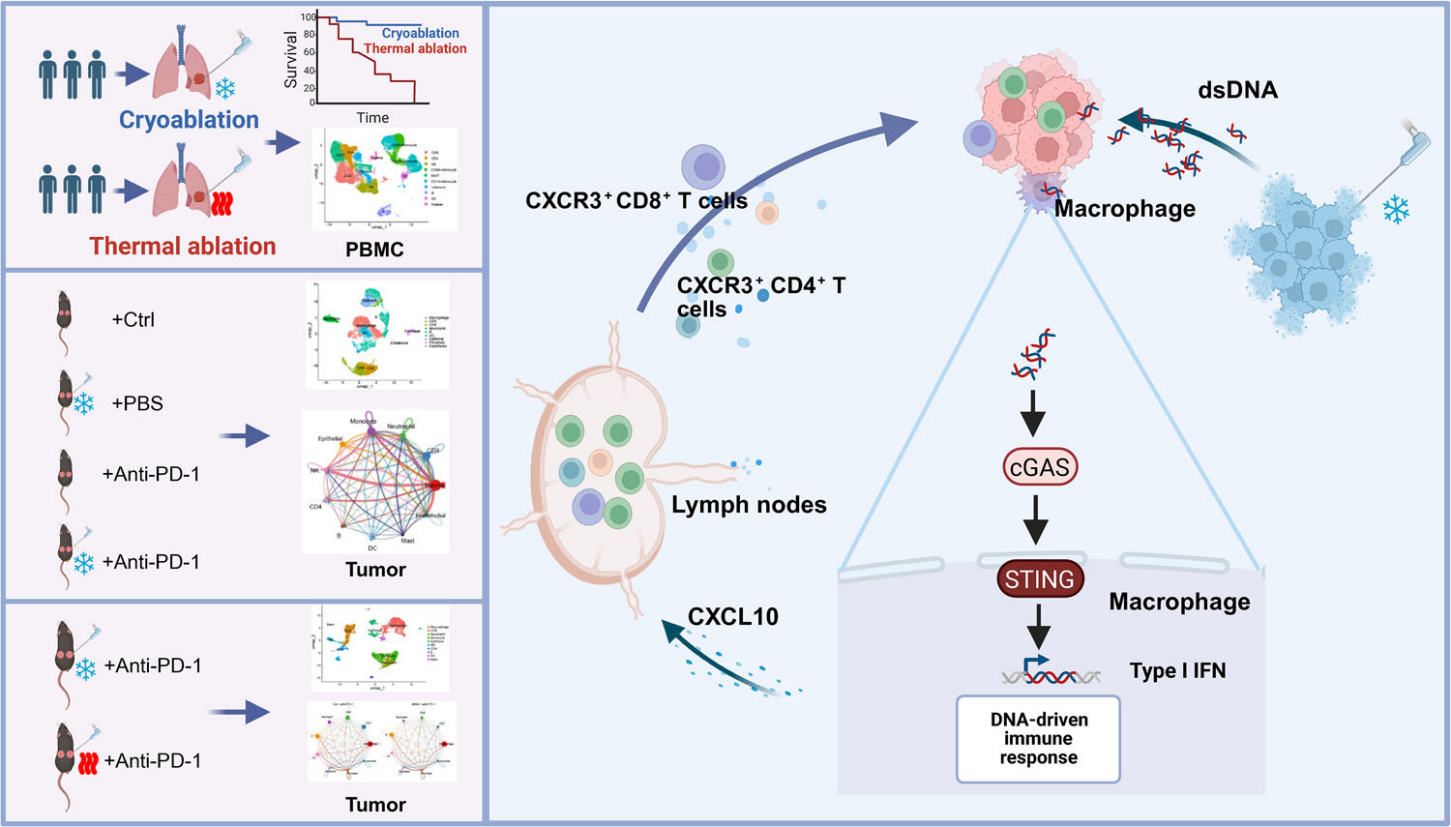
Mechanism schematic of cryoablation activating the cGAS–STING signaling pathway in macrophages. In patients with oligo‐residual disease undergoing cryoablation and thermal ablation after immunotherapy, in mouse models comparing CA + anti‐PD‐1 with MWA + anti‐PD‐1 or with anti‐PD‐1 alone, scRNA‐seq consistently showed that cryoablation enhances macrophage activation and anti‐tumor efficacy. Mechanistically, cryoablation‐induced tumor cell lysis releases dsDNA that is engulfed by macrophages, triggering cGAS–STING signaling and secretion of CXCL10. This chemokine recruits CXCR3^+^ CD8^+^ and CXCR3^+^ CD4^+^ T cells from draining lymph nodes into the tumor microenvironment to mount anti‐tumor immunity. CA, cryoablation; MWA, microwave ablation.

In conclusion, we found that cryoablation‐induced dsDNA release could activate the cGAS‐STING pathway in macrophages, driving expansion of CXCL10^+^ macrophages, which recruit CXCR3^+^ T cells from draining lymph nodes into the tumor microenvironment, and thereby exert a systemic antitumor efficacy (Figure 8). Combining cryoablation with a STING agonist further amplified the therapeutic efficacy of an‐PD‐1 treatment. Our findings might offer a novel treatment strategy for driver‐gene‐negative advanced NSCLC.

## 4 Methods

### 4.1 Enrollment of Patients

Advanced NSCLC patients with oligo‐residual disease after immunotherapy who underwent cryoablation or thermal ablation at Shanghai Pulmonary Hospital between July 2023 and June 2025 were enrolled in this study. The inclusion criteria included: age ≥ 18 years; histologically or cytologically confirmed stage IIIB–IV NSCLC; Anti‐PD‐1/PD‐L1 monotherapy before ablation displayed the best response of stable disease (SD) or partial response (PR); Eastern Cooperative Oncology Group performance status (ECOG‐PS) ≤2; and complete electronic medical records. Patients were excluded if they had ECOG PS > 2, experienced systemic progression on prior anti‐PD‐1/PD‐L1 monotherapy, received <2 cycles of immunotherapy, or were lost to follow‐up. This study was approved by the Ethics Committee of Shanghai Pulmonary Hospital (L20‐244), and informed consent forms were obtained from all enrolled patients.

### 4.2 Cell culture

The *Kras^G12D/+^Tp53^−/−^* (KP), *Kras^G12D/+^Tp53^−/−^* female (KP‐F), and *Kras* ^G12D/+^ *Lkb1* ^−/−^ (KL) GEMM‐derived primary cell lines were generated in our laboratory as previously described [40, 41]. The Lewis lung cancer (LLC), and RAW264.7, PC‐9, and THP‐1 cell lines were obtained from the National Collection of Authenticated Cell Cultures. LLC and RAW264.7 cells were maintained in DMEM (Gibco, #C11995500BT) supplemented with 10 % fetal bovine serum (Gibco, #10099), 100 U/mL penicillin, and 100 mg/L streptomycin (Gibco, #15070063). KP, KP‐F, KL, PC‐9, and THP‐1 cells were cultured in RPMI‐1640 medium (Gibco, #C11875500BT) containing the same supplements. All cell lines were incubated at 37°C with 5 % CO_2_ in a humidified atmosphere.

### 4.3 Mice

In vivo experiments were performed using 6‐ to 8‐week‐old male C57BL/6 mice (Shanghai Slack Laboratory Animal Co., Ltd., Shanghai, China), female *Rag2* ^−/−^ mice, and female *Cxcr3^−/−^* mice (Shanghai Model Organisms Center, Inc., Shanghai, China). A total of 1 × 10^6^ KP, 1 × 10^6^ KP‐F, 5 × 10^5^ LLC, or 5 × 10^5^ KL cells were injected subcutaneously into the bilateral flanks of the mice. When tumors reached 150–400 mm^3^ (calculated as width × length^2^ / 2), mice were randomized to receive ablation or drug treatment. All mice were housed under SPF conditions with a 12‐h light/dark cycle, 18–22°C ambient temperature, and 50–60 % humidity. This study was approved by the Institutional Committee for Animal Care and Use (K25‐038Y), Shanghai Pulmonary Hospital, and conducted in accordance with institutional guidelines.

### 4.4 Tumor Ablation in Mice

Cryoablation was performed on mouse tumors using the AI Epic S40 combined cryo‐thermal ablation system equipped with a 1.7‐mm‐diameter cryo‐thermal probe (RCS17; Hygea Medical Technology Co., Ltd.). The probe was inserted into the tumor, cooled to −140°C for 1 min, rewarmed to 40°C, and the freeze–thaw cycle was repeated once. Thermal ablation was performed using an MTC‐3 microwave therapeutic equipment equipped with a 1.3‐mm water‐cooled antenna (MTC‐3CA‐II/3; Vision Medical Technology Co., Ltd., China) at 5 W for 3 min. All ablation was performed exclusively on the right‐sided tumor of mice.

### 4.5 In Vivo Drug Administration Experiments

Anti‐PD‐1 (HRP00262) provided by Hengrui Medicine Co., Ltd. was administered intraperitoneally at 10 mg kg^−^^1^ every two days. FTY720 (SML0700; Sigma‐Aldrich) was given by daily oral gavage at 2 mg kg^−^^1^. Clodronate liposomes (40337ES10; Yeasen) or control liposomes (40338ES10; Yeasen) were injected intraperitoneally at 200 µL per mouse every three days. For T cell depletion, anti‐mouse CD8α (Selleck, A2102), anti‐mouse CD4 (Selleck, A2101), or IgG2b isotype control (Selleck, A2116) was administered intraperitoneally at 200 µL per mouse. For NK cell depletion, anti‐mouse NK1.1 (BioXcell, BE0036) or IgG2a isotype control (BioXcell, BE0089) was administered intraperitoneally at 200 µg per mouse every three days (total of five doses). AMG487 (S8682, Selleck, Houston, USA) was administered subcutaneously at 5 mg/kg twice daily for 10 consecutive days. DiABZI (S8796, Selleck, Houston, USA) was delivered via tail‐vein injection at 3 mg/kg every 3 days for a total of five doses.

### 4.6 10X Genomics Single‐Cell RNA Sequencing

#### 4.6.1 Preparation of PBMC

Peripheral blood was collected in EDTA‐anticoagulated tubes 14 days post‐ablation. PBMCs were isolated by density‐gradient centrifugation using Ficoll‐Paque (GE, #17‐1440‐02) according to the manufacturer's instructions.

#### 4.6.2 Preparation of Tumor Tissue for Single‐Cell Sequencing

The freshly collected tumor tissues were washed twice with pre‐cooled RPMI 1640 medium containing 0.04% BSA. The tissues were then mechanically dissociated into approximately 0.5 mm^3^ fragments using surgical scissors and transferred into freshly prepared enzymatic digestion solution. The digestion mixture contained RPMI 1640 (Conring, 10‐040‐CVR), 0.04% BSA (MACS, 1000076), and 0.2% collagenase II (Gibco, 17101015). The samples were incubated at 37°C for 30–60 min with gentle inversion every 5–10 min. The digested cell suspension was filtered through a BD 40 µm cell strainer (Falcon, 352340) 1‐2 times. The filtrate was centrifuged at 300 × g for 5 min at 4°C. The cell pellet was resuspended in appropriate medium, mixed with an equal volume of red blood cell lysis buffer (Miltenyi, 130‐094‐183), and incubated at 4°C for 10 min. After centrifugation at 300 × g for 5 min, the supernatant was discarded. The pellet was washed once with medium, followed by another centrifugation at 300 × g for 5 min, and the final supernatant was removed. In the four‐group comparison (Ctrl, anti‐PD‐1, CA, and CA + anti‐PD‐1), CD45^+^ immune cells were enriched from single‐cell suspensions using mouse CD45 MicroBeads (Miltenyi, 130‐052‐301) according to the manufacturer's protocol. In the comparative experiment between CA plus anti‐PD‐1 and MWA plus anti‐PD‐1, the entire tumor‐derived single‐cell suspension was used directly for subsequent sequencing without CD45 enrichment. Finally, the cells were re‐suspended with 1ml RPMI 1640 medium (Conring, 10‐040‐CVR) with 0.04% BSA added. Single‐cell suspension concentration and cell viability were then evaluated by Luna‐FL cell counter (Logos Biosystems, Korea).

#### 4.6.3 Construction of scRNA‐seq Library

The freshly prepared single‐cell suspension was adjusted to a concentration of 700–1200 cells/µL. Library preparation and loading were performed following the manufacturer's protocol for the 10×Genomics Chromium Next GEM Single Cell 3ʹ Reagent Kits v3.1 (cat.no.PN‐1000268). The constructed libraries were sequenced on the Illumina Nova 6000 PE150 platform for high‐throughput sequencing.

#### 4.6.4 ScRNA‐seq Data Processing

The FASTQ files were processed and aligned to the CRCm3 reference genome using Cell Ranger software (version 8.0.1) from 10x Genomics, with unique molecular identifier (UMI) counts summarized for each barcode. The UMI count matrix was then analyzed using the Seurat package [42] (version 4.0.0). For filtering, low‐quality cells and likely multiplet captures were removed according to the following criteria: Cells were filtered by (1) gene numbers < 200, (2) UMI <1,000, (3) log10GenesPerUMI < 0.7, (4) percentage of mitochondrial RNA UMIs > 20%, and (5) percentage of hemoglobin RNA UMIs > 10%. Subsequently, the DoubletFinder package (version 2.0.3) was used to identify potential doublets. To obtain the normalized gene expression data, library size normalization was processed using the NormalizeData function. Specifically, the global‐scaling normalization method “LogNormalize” normalized the gene expression measurements for each cell by the total expression, multiplied by a scaling factor (10,000 by default), and log‐transformed the results.

Non‐linear dimensionality reduction using Uniform Manifold Approximation and Projection (UMAP) was applied to generate 2D visualizations. Cell identities were annotated based on marker genes cataloged in the CellMarker database and documented in the published literature. Differences in the relative abundance of cell subsets across groups were evaluated using the Kruskal–Wallis test. To explore the immune‐related biological functions of differentially expressed genes, we performed KEGG pathway enrichment analysis using clusterProfiler. Additionally, cell–cell interactions and receptor–ligand pairs among all major cell types were inferred using CellChat.

#### 4.6.5 Luminex Assay

Whole blood of patients and mice was centrifuged at 3000 × g for 20 min at 4°C, and the supernatant was collected. 50ul of sample, magnetic beads, standard, and quality‐control solution were added to each well of a Luminex plate (Bio‐Rad, 12009159, 171AK99MR2) and incubated for 1 h at RT. After three washes with assay buffer, 50 µL of the biotinylated detection antibody provided in the kit was added and incubated for 30 min at RT. Following three additional washes, 50 µL of streptavidin–phycoerythrin was added and incubated for 20 min at RT. The plate was then washed three times with 200 µL wash buffer, resuspended in 100 µL sheath fluid, incubated for 2 min, and read on a Luminex instrument (Luminex‐200). Data were acquired and analyzed using Milliplex Analyst 5.1 software.

#### 4.6.6 Flow Cytometry

To characterize the immune microenvironment KP and LLC tumor tissues, freshly resected tumors were mechanically minced and enzymatically digested with the Mouse Tumor Dissociation Kit (Miltenyi Biotec, 130‐096‐730) following the manufacturer's protocol. The resulting suspension was passed through a 70‐µm strainer, and cells were pelleted by centrifugation (500 × g, 5 min, 4°C). Erythrocytes were removed by incubation in red‐blood‐cell lysis buffer (Tiangen, GRT122‐02) for 5 min at room temperature (RT). After two washes with PBS (Biosharp, BL30‐2A) and one wash with DPBS (Biosharp, BL310‐A), live/dead discrimination was performed by staining with Fixable Viability Dye (Thermo Fisher, 65‐2860‐40) for 15 min at RT in the dark. Cells were then washed once with stain buffer (BD Biosciences, 554657) and blocked with anti‐mouse CD16/CD32 (BD Pharmingen, 553142) for 10 min at RT before addition of fluorochrome‐conjugated antibodies. Surface markers were stained for 30 min at RT and then permeabilized using Transcription Factor Buffer Set (BD Pharmingen, 562574), and finally incubated with antibodies against intracellular markers for 30 min at RT.

To characterize the immune microenvironment of patients' PBMCs, peripheral blood was collected in EDTA‐anticoagulated tubes before and 14 days post‐ablation. PBMCs were isolated by density‐gradient centrifugation using Ficoll‐Paque (GE, #17‐1440‐02) according to the manufacturer's instructions. After washing once with DPBS, live/dead discrimination was performed by staining with Fixable Viability Dye (Thermo Fisher, 65‐2860‐40) for 15 min at room temperature. Cells were then washed once with stain buffer (BD Pharmingen, 554657) and blocked with anti‐human CD16/CD32 (BD Pharmingen, 564219) for 10 min at room temperature. Subsequently, cells were stained with the indicated cell‐surface markers, fixed/permeabilized using the Transcription Factor Buffer Set (BD Pharmingen, 562574), and finally stained with the indicated intracellular marker antibodies. Cells were acquired on Cytek Aurora (Cytek Biosciences) and analyzed with FlowJo software (version 10.9.0).

#### 4.6.7 Flow Antibodies

The subcutaneous tumor‐infiltrating tumor, PBMC, splenocytes and LN of mice were stained with fluorochrome‐conjugated antibodies against mouse CD45 (BD Pharmingen, 557659), CD3 (BD Pharmingen, 562286), CD4 (BD Pharmingen, 560468), CD8a (BD Pharmingen, 46‐0081‐82), CD8a (BD Pharmingen, 551162), CXCR3 (Thermo Fisher, 12‐1831‐82), CD11b(Biolegend, 101233), F4/80 (BD Pharmingen, 743282), NKp46 (BD Pharmingen, 564069), CD69 (BD Pharmingen, 566501), CD86 (BD Pharmingen, 558703), CD206 (BD Pharmingen, 568273), CD44 (Biolegend, 103040), CD62L (Biolegend, 104453), CXCL10 (Bioss, bs‐1502R‐APC). The PBMC of humans were stained with fluorochrome‐conjugated antibodies against human CD3 (BD Pharmingen, 561800), CD4 (BD Pharmingen, 562402), CD8 (BD Pharmingen, 560662), CXCR3 (BD Pharmingen, 353729), and IFN‐γ (BD Pharmingen, 506541).

#### 4.6.8 Hematoxylin and Eosin (H&E) Staining

Paraffin‐embedded tissue sections (4 µm) were deparaffinized in xylene and rehydrated through a graded ethanol series. Sections were stained with hematoxylin (Servicebio, G1004) for 5 min, rinsed in running tap water, differentiated in 1% acid alcohol for 10 s, and blued in ammonia water. After eosin (Servicebio, G1001) staining for 2 min, sections were dehydrated through an ascending ethanol series, cleared in xylene, and mounted with neutral balsam (Servicebio, G1403). Images were acquired using a Pannoramic MIDI digital slide scanner (Servicebio).

#### 4.6.9 Immunohistochemistry (IHC)

After paraffin embedding of the mice tumors, 4 µm sections were cut and mounted on glass slides. Slides were deparaffinized in xylene, rehydrated through absolute ethyl alcohol, and transferred to citrate‐based antigen retrieval buffer (Servicebio, G1202). Antigen retrieval was done by microwaving at medium power for 8 min, resting for 8 min, and microwaving again for 8 min. After cooling at RT, slides were washed three times in PBS on a shaker. Endogenous peroxidase was blocked with 3 % H_2_O_2_ (Servicebio, G0115) for 25 min at RT in the dark, followed by 30 min blocking with 3 % BSA (Servicebio, G5001) to prevent non‐specific binding. Sections were incubated overnight at 4°C with primary antibodies against CD8 (Servicebio, GB15068, 1:400), CD4 (Servicebio, GB15064, 1:200), and CD68 (Servicebio, GB113109, 1:200). For negative controls, the primary antibody was replaced with isotype‐matched IgG under identical conditions. After 1 h incubation at 37°C with secondary antibody (Servicebio, GB23301), slides were washed three times with PBS and developed with DAB substrate (Servicebio, G1211). Sections were counterstained with hematoxylin (Servicebio, G1004) for 1–2 min at RT, mounted with neutral resin (Servicebio, G1403), and scanned using the Pannoramic MIDI digital slide scanner (Servicebio). Positive‐cell density on the whole‐slide images was quantified with Aipathwell software (Servicebio).

#### 4.6.10 Immunofluorescence (IF)

Paraffin sections were deparaffinized and rehydrated, subjected to antigen retrieval, and then cooled to RT. After blocking with 3 % BSA (Servicebio, GC305010) for 30 min, the sections were incubated overnight at 4°C with a cocktail of primary antibodies against mouse dsDNA (Sigma‐Aldrich, ZMS1047, 1:200) and F4/80 (Servicebio, GB113373, 1:500). Following three PBS washes, species‐appropriate fluorophore‐conjugated secondary antibodies were applied and incubated for 50 min at RT in the dark. Nuclei were counterstained with DAPI. Fluorescence images were acquired on a Nikon Eclipse C1 microscope and analyzed using ImageJ v1.53.

#### 4.6.11 Generation of Primary Bone Marrow‐Derived Macrophages (BMDMs)

Mice were soaked in 70% ethanol for 5 min. Under aseptic conditions, the femurs and tibias were harvested, and the attached muscles were carefully removed. Bone marrow cells were flushed out with ice‐cold PBS using a syringe fitted with a 25‐gauge needle. The collected bone marrow suspension was passed through a 70 µm cell strainer to remove cell clumps. After red blood cell lysis using lysis buffer (Tiangen, RT122‐02), the bone marrow cells were plated in non‐tissue culture‐treated dishes and cultured in DMEM (Gibco, #C11995500BT) supplemented with 10% FBS, 1% penicillin‐streptomycin, and 20 ng/mL M‐CSF (MCE, HY‐P7085) at 37°C in a humidified incubator with 5% CO_2_. Mature BMDMs were harvested on days 5–7.

#### 4.6.12 Adoptive Transfer Cells

For BMDM adoptive transfer experiments, primary BMDMs were generated as described above. BMDMs were transduced with control or CXCL10‐knockout (CXCL10‐KO) lentiviruses (Tsingke, China) in the presence of Polybrene (MCE, HY‐112735). Following 24 h of culture, CXCL10‐WT and CXCL10‐KO BMDMs were harvested. For T cell adoptive transfer experiments, splenic T cells from wild‐type (WT) and CXCR3‐knockout (CXCR3‐KO) mice were isolated using the EasySep Mouse T Cell Isolation Kit (STEMCELL, 19851). A total of 1 × 10^5^ T cells were resuspended in 100 µL PBS and administered to recipient mice via tail vein injection, initiated on the day of anti‐PD‐1 treatment and repeated every 3 days for a total of three injections. Tumor growth was monitored and recorded throughout the treatment course. Recipient mice were sacrificed at day 15 post‐treatment initiation, and tumor weights were subsequently measured and analyzed.

#### 4.6.13 In Vitro Stimulation Assays

For in vitro stimulation assays, RAW264.7, BMDM, and TPH1 cells were plated at 2 × 10^6^ per well in 6‐well plates. KP and PC‐9 cells were cultured in 10 cm dishes, detached with trypsin (Gibco, 25200072), collected by centrifugation, and resuspended in PBS (Servicebio, G4202). The suspension was subjected to two freeze–thaw cycles (liquid nitrogen for 2 min, 37°C for 3 min each). After centrifugation, the supernatant was transferred to RAW264.7, BMDM, or TPH1 cells using Lipofectamine 2000 (Invitrogen, 11668019). To block the cGAS–STING pathway, 10 nm STING‐IN‐2 (MCE, HY‐138682) was added immediately after transfection. Cells were cultured for an additional 48 h before downstream analyses.

#### 4.6.14 dsDNA Detection

Mouse serum was collected via retro‐orbital bleeding at 7 days post‐ablation. For tumor cell lysates, two freeze–thaw cycles (liquid nitrogen for 2 min, 37°C for 3 min each) were applied to mimic cryoablation, and water bath heating (60°C for 5 min) was used to simulate thermal ablation. The concentration of dsDNA was determined using the dsDNA HS Assay Kit (Yeasen,12640ES60) and measured with a Qubit 4 Fluorometer (Thermo Fisher, Q33238).

#### 4.6.15 Enzyme‐Linked Immunosorbent Assay (ELISA)

CXCL10 levels in the supernatants of RAW264.7 and BMDM cells were measured using a mouse CXCL10 ELISA kit (Jonlnbio, JL13372) according to the manufacturer's instructions. CXCL10 in THP‐1 cell supernatants was detected using a human CXCL10 ELISA kit (Jonlnbio, JL11028). Briefly, 100 µL of samples and different concentrations of standards were added into the corresponding wells and incubated at 37°C for 1 h. Then the cells were incubated with 100 µL of biotinylated detection antibody working solution at 37°C for 60 min. Each well was then washed three times with 300 µL 1X wash buffer, followed by the addition of 100 µL enzyme‐conjugate working solution and a further 30 min incubation at 37°C. The plate was washed five times with 1× wash buffer, and 90 µL TMB substrate was added to each well. After 15 min incubation at 37°C in the dark, 50 µL stop solution was added to each well, and the absorbance at 450 nm was immediately read on the Varioskan Flash microplate reader (Thermo Fisher).

#### 4.6.16 Quantitative RT‐PCR

Total RNA was extracted from RAW264.7, THP‐1, and BMDM cells using RNA Fast200 (Fastagen, 220010) following the manufacturer's instructions, and cDNA was synthesized with PrimeScript RT Master Mix (Takara, RR036A) on a T100 Thermal Cycler (Bio‐Rad). Quantitative RT‐PCR was carried out with TB Green Premix Ex Taq II (Takara, RR820A) on an Mx3000P Real‐Time PCR System (Agilent Technologies/Stratagene). Gene transcripts were normalized to GAPDH as the internal control. Primer sequences for RAW264.7 and BMDM cells are listed below: Gapdh‐Forward:CATCACTGCCACCCAGAAGACTG; Gapdh‐Reverse: ATGCCAGTGAGCTTCCCGTTCAG. Cxcl10‐Forward: ATCATCCCTGCGAGCCTATCCT; Cxcl10‐ Reverse: GACCTTTTTTGGCTAAACGCTTTC. Primer sequences for THP‐1 cells are listed below: Gapdh‐Forward: AGCCACATCGCTCAGACAC; Gapdh‐Reverse: GCCCAATACGACCAAATCC. Cxcl10‐Forward: CTGCCATTCTGATTTGCTGCC; Cxcl10‐ Reverse: AATGCTGATGCAGGTACAGCG.

#### 4.6.17 Western Blot

In the KP tumor model, mice underwent cryoablation or microwave ablation. At 24 h post‐ablation, the ablated tumor tissues were harvested for the detection of apoptosis, necrosis, and pyroptosis biomarkers. At day 14 post‐ablation, the contralateral tissues of ablation were collected for cGAS‐STING signaling pathway analysis. Cells were lysed on ice for 30 min with RIPA buffer (Beyotime, P0013B) supplemented with protease and phosphatase inhibitors (Epizyme, GRF101). Protein concentrations were determined with the BCA assay (Epizyme, ZJ102) on a Thermo Fisher Scientific Varioskan Flash plate reader. Samples were mixed with loading buffer (Epizyme, LT101), boiled for 10 min, and 10 µg total protein was separated by SDS‐PAGE. Proteins were transferred to PVDF membranes (Millipore, ISEQ00010), blocked with protein‐free blocking solution (Epizyme, PS108), and incubated overnight at 4°C with primary antibodies against mouse cGAS (Cell Signaling Technology, #31659, 1:1000), human cGAS (Proteintech, 29958‐1‐AP, 1:40000), STING (Cell Signaling Technology, #13647, 1:1000), phospho‐STING (Cell Signaling Technology, #72971, 1:1000), TBK1 (Cell Signaling Technology, #3504, 1:1000), phospho‐TBK1 (Cell Signaling Technology, #5483, 1:1000), β‐actin (Cell Signaling Technology, #4970, 1:1000), Cleaved Caspase 3 (Proteintech, 25128‐1‐AP, 1:1000), phospho‐MLKL (Cell Signaling Technology, #37333, 1:1000), GSDMD‐N (Abclonal, A20197 1:1000). After three times of TBST (Epizyme, PS103S) washes, membranes were incubated with anti‐rabbit IgG secondary antibody (Cell Signaling Technology, #7074, 1:2000) for 1h at RT. Following three times of TBST washes (Epizyme, PS103S), chemiluminescence was detected using an ECL substrate (Epizyme, SQ201) on the Varioskan Flash reader. Western blot images were analyzed with ImageJ v1.53.

### 4.7 Statistical Analysis

All statistical analyses and data visualizations were performed using R version 4.5.1, GraphPad Prism 9.5.1, and IBM SPSS Statistics 26. Normality and homogeneity of variances were first assessed with the Shapiro–Wilk test and Levene's test, respectively. Depending on the data distribution, differences between two groups were evaluated with Student's *t* test, Welch's t test, or the Mann–Whitney U test. Comparisons among three or more groups were carried out using one‐way ANOVA or the Kruskal–Wallis H test. Associations between variables were examined with Pearson's correlation analysis. Survival analysis was performed using the Kaplan‐Meier method. *P* < 0.05 was considered statistically significant.

## Author Contributions

X.Z. conceived and designed the study, collected clinical data, prepared single‐cell sequencing samples, performed in vitro and in vivo experiments and immune‐microenvironment analyses, conducted statistical analyses, wrote and revised the manuscript. Z.X. conducted single‐cell data analysis, experimental method guidance, and wrote and revised the manuscript. L.L. collected clinical data, prepared single‐cell sequencing samples, performed in vitro and in vivo experiments, and wrote and revised the manuscript. J.W., X.L., J.Y., J.Y., H.S., G.G., L.W., and X.C. contributed to clinical data collection and manuscript writing. B.C. and Y.L. participated in clinical data collection. F.L. contributed to research conceptualization. L.W. contributed to technical support and manuscript revision. S.Y. contributed to research conceptualization, technical support, and manuscript revision. S.R. conceived and designed the study, supervised the overall research direction, and revised the manuscript.

## Funding

This work was supported by the National Key Research and Development Program (2023YFC2414000), Noncommunicable Chronic Diseases‐National Science and Technology Major Project (2024ZD0520200, 2024ZD0520206), and the National Natural Science Foundation of China (82203046, 82372794, 82572985).

## Ethics Approval Statement

This study was approved by the Ethics Committee of Shanghai Pulmonary Hospital (L20‐244), and informed consent forms were obtained from all enrolled patients.

## Conflicts of Interest

All authors declared no conflict of interest.

## Supporting information




**Supporting File**: advs74786‐sup‐0001‐SuppMat.docx.

## Data Availability

The data that support the findings of this study are available from the corresponding author upon reasonable request.;
