# Supplementary material for: Cryoablation Activates the cGAS–STING‐CXCL10 Axis in Macrophages to Enhance Anti‐Tumor Immunity in NSCLC
Source: Adv Sci (Weinh). 2026 Mar 12;13(29):e21931. doi: 10.1002/advs.202521931 (PMC13205896; doi:10.1002/advs.202521931)
Supplement: Supplementary file 1 — Supporting File: advs74786‐sup‐0001‐SuppMat.docx. [file ADVS-13-e21931-s001.docx]

**Cryoablation Activates the cGAS–STING-CXCL10 Axis in Macrophages to Enhance Anti-tumor Immunity in NSCLC**

Xinxin Zhi^1#^, Zhengcao Xing^1#^, Libo Luo^1#^, Jiale Wang^1^, Xinyu Liu^1^, Jia Yu^1^, Jizhong Yin^1^, Bin Chen^1^, Yiwei Liu^1^, Hui Sun^1^, Guanghui Gao^1^, Lei Wang^1^, Xiaoxia Chen^1^, Fei Li^2^, Hu Ma^3*^, Lin Wang^1,2*^, Shuo Yang^1*^, Shengxiang Ren^1*^

^#^ Xinxin Zhi, Zhengcao Xing and Libo Luo contributed equally to this study.

^1^ Department of Medical Oncology, Shanghai Pulmonary Hospital, School of Medicine, Tongji University, Shanghai, China.

^2^ Department of Medical Oncology, Shanghai Pulmonary Hospital, School of Medicine, Tongji University, Shanghai, China.

^3^ Department of Oncology, The Second Affiliated Hospital of Zunyi Medical University, Zunyi, China.

***Corresponding authors:**

Shengxiang Ren, Department of Medical Oncology, Shanghai Pulmonary Hospital, School of Medicine, Tongji University, Shanghai, 200433, China. Tel: +86-21-65115006; E-mail: harry_ren@tongji.edu.cn

Shuo Yang, Department of Medical Oncology, Shanghai Pulmonary Hospital, School of Medicine, Tongji University, Shanghai, China. E-mail: syang0213@foxmail.com

Lin Wang, Department of Medical Oncology, Shanghai Pulmonary Hospital, School of Medicine, Tongji University, Shanghai, China; Department of Pathology and Frontier Innovation Center, School of Basic Medical Sciences, Fudan University, Shanghai, China. E-mail: [lin_wang_@fudan.edu.cn](mailto:lin_wang_@fudan.edu.cn)

Hu Ma, Department of Oncology, The Second Affiliated Hospital of Zunyi Medical University, Zunyi, China. Email: mahuab@163.com

Table S1. Clinicopathological characteristics of patients enrolled.

| Characteristics | Thermal ablation (n, %) | Cryoablation (n, %) | p-value |
| --- | --- | --- | --- |
| **Gender** |  |  |  |
| Male | 44 (84.6%) | 26 (100%) | 0.086 |
| Female | 8 (15.4%) | 0 (0%) |  |
| **Age (years)** |  |  |  |
| Mean (SD) | 67.5 (7.29) | 66.3 (9.95) | 0.591 |
| **Histology** |  |  |  |
| Squamous carcinoma | 21 (40.4%) | 9 (34.6%) | 0.322 |
| Adenocarcinoma | 27 (51.9%) | 12 (46.2%) |  |
| NSCLC | 4 (7.7%) | 5 (19.2%) |  |
| **PD-L1_status** | |  |  |
| <1% | 26 (50.0%) | 7 (26.9%) | 0.071 |
| 1-49% | 8 (15.4%) | 5 (19.2%) |  |
| ≥50% | 13 (25.0%) | 6 (23.1%) |  |
| Unknown | 5 (9.6%) | 8 (30.8%) |  |
| **TNM** |  |  |  |
| III | 9 (17.3%) | 10 (38.5%) | 0.076 |
| IV | 43 (82.7%) | 16 (61.5%) |  |
| **Prior lines of therapy** |  |  |  |
| First-line | 41 (78.8%) | 23 (88.5%) | 0.465 |
| Later-line | 11 (21.2%) | 3 (11.5%) |  |
| **ECOG PS** |  |  |  |
| 0-1 | 39 (75.0%) | 22 (84.6%) | 0.497 |
| 2 | 13 (25.0%) | 4 (15.4%) |  |
| **Smoking status** | |  |  |
| Never smoked | 41 (78.8%) | 22 (84.6%) | 0.761 |
| Current of former smoker | 11 (21.2%) | 4 (15.4%) |  |

**
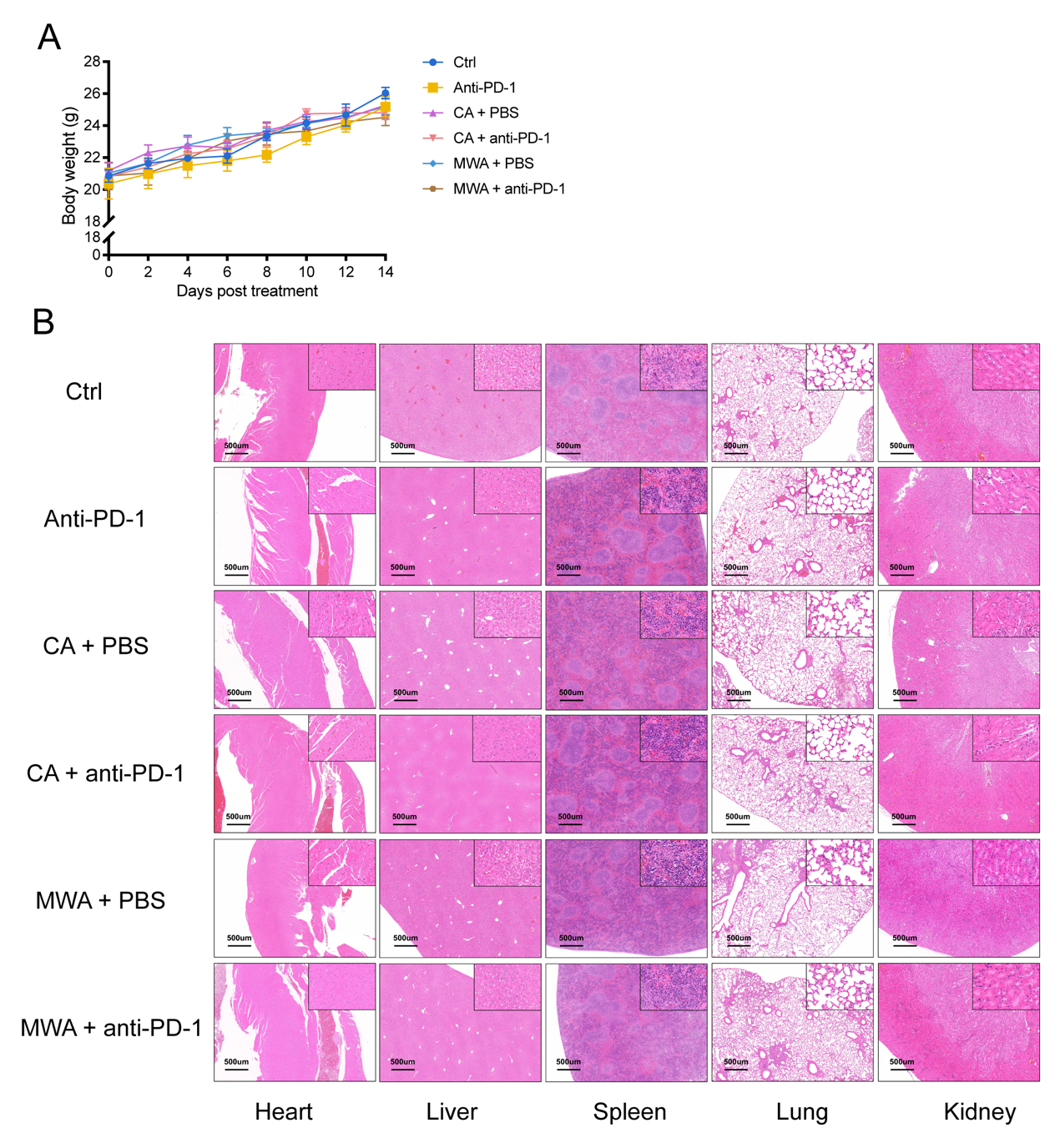
**

**Figure S1. Body weight change after ablation or immunotherapy of mice**
A, C57BL/6 mice bearing bilateral KP tumors were randomized to six groups—control, anti-PD-1, cryoablation (CA) + PBS, microwave ablation (MWA) + PBS, MWA + anti-PD-1, or CA + anti-PD-1—and body weight was recorded every 2 days (n=5/group). B, H&E staining of the heart, liver, spleen, lung, and kidney from KP tumor-bearing mice in the respective groups at 14 days post-treatment. Data are calculated using one-way ANOVA and presented as mean ± SEM; ns, not significant.


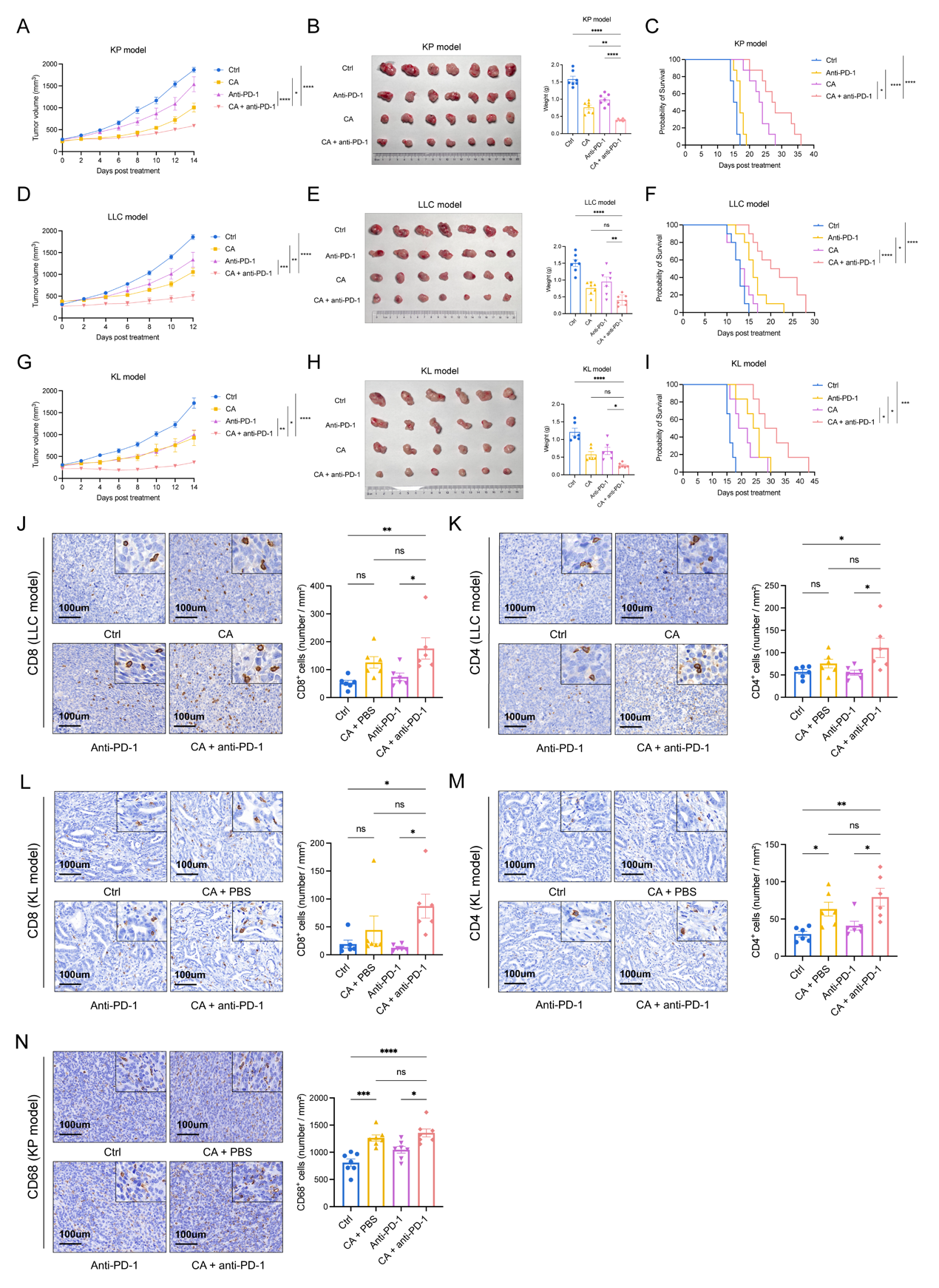


**Figure S2.** **Antitumor efficacy and intra-tumoral immune response to cryoablation combined with anti–PD-1 therapy**

A-C, KP model: tumor growth curves (A), representative tumor size (B left) and weight (B right), and survival analysis (C) after control, cryoablation (CA), anti–PD-1, or CA + anti–PD-1 treatment (n = 7 for growth; n = 8 for survival). D–F, LLC model: growth curves (D), tumor size (E left) and weight (E right), and survival (F) (n = 7 for growth; n = 10 for survival). G–I, KL model: growth curves (G), tumor size (H left) and weight (H right), and survival (I) (n = 6 for both growth and survival). J, Representative IHC images (left) and quantification (right) of CD8⁺ T cells in LLC tumors 12 days post-treatment. K, Representative IHC images (left) and quantification (right) of CD4⁺ T cells in the LLC tumors. L, Representative IHC images (left) and quantification (right) of CD8⁺ T cells in KL tumors 14 days post-treatment. M, Representative IHC images (left) and quantification (right) of CD4⁺ T cells in the KL tumors. N, Representative IHC images (left) and quantification (right) of CD68⁺ T cells in the KP tumors. A, B, D, E, G, H, J-N were calculated using one-way ANOVA. *The p* values of C, F and I were determined by log-rank test (Mantel-Cox). Data are presented as mean ± SEM; ns, not significant; **p*<0.05, ***p*<0.01, ****p*<0.001, *****p*<0.0001. SEM, standard error of the mean.


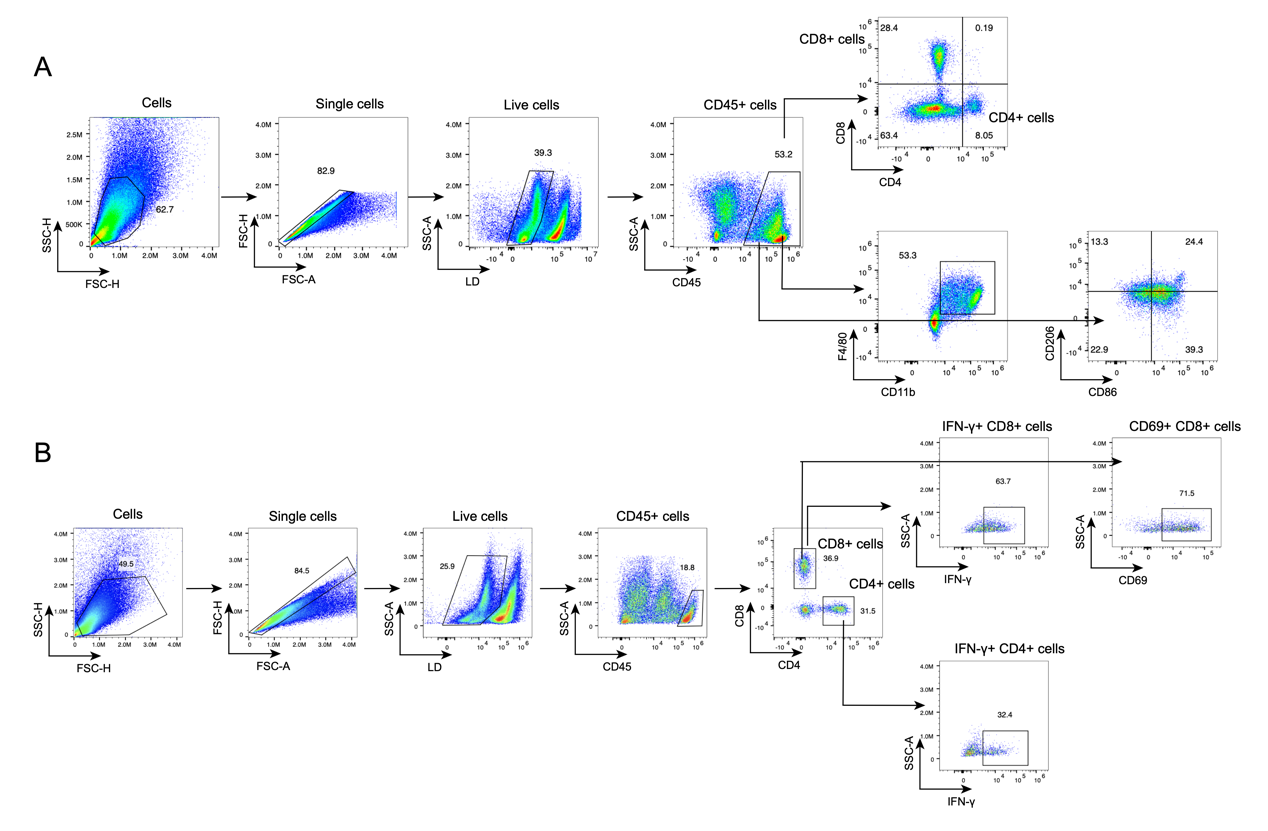

**Figure S3. Flow-cytometry gating strategies for tumor-infiltrating T cells**A, Gating scheme for KP tumor CD4⁺ and CD8⁺ T cell subpopulations.
B, Gating strategy for LLC tumor infiltrating T cell subsets.


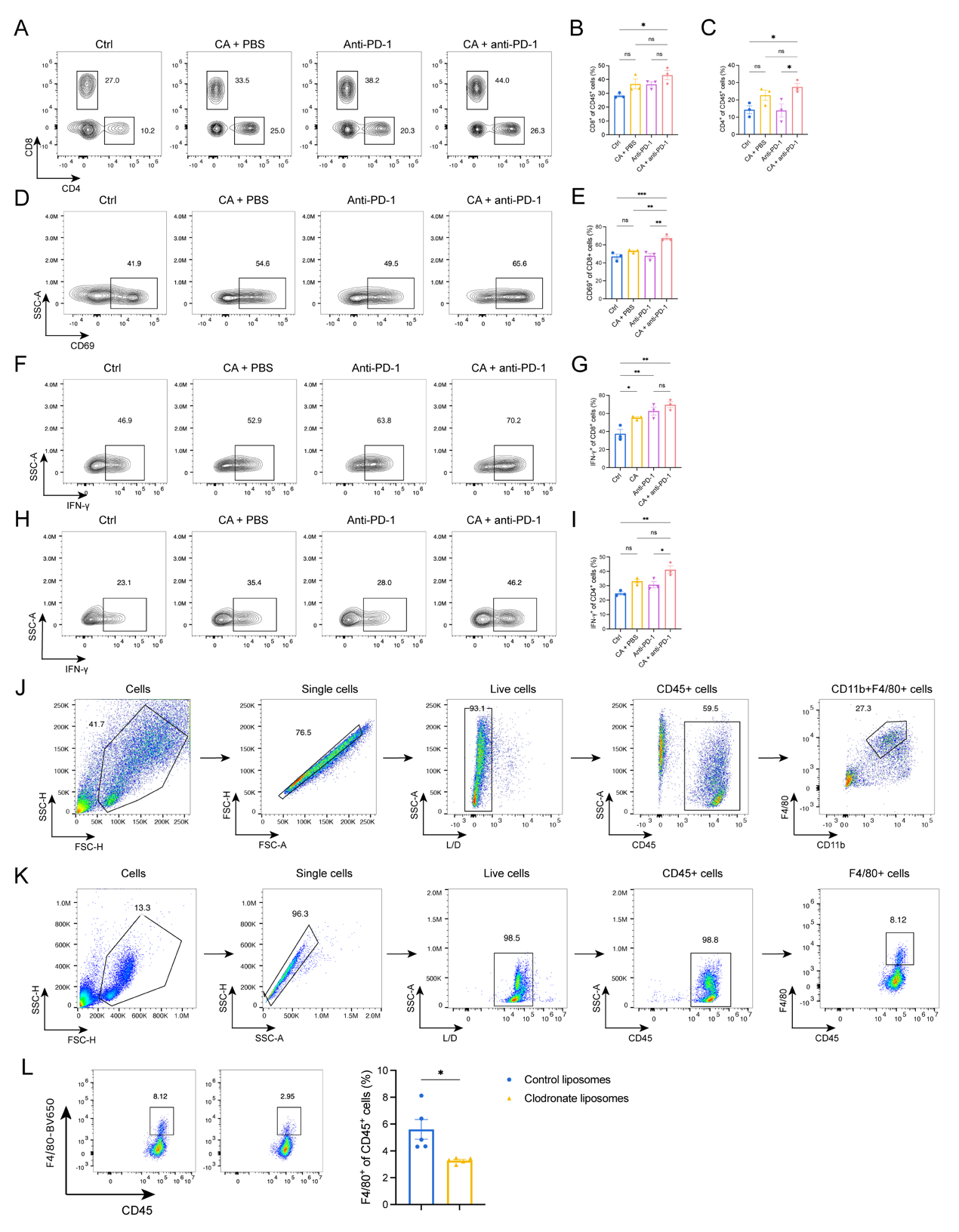


**Figure S4. Alterations of T cells abundance in the tumor microenvironment after cryoablation and immunotherapy**
A, Representative fluorescence images of tumor-infiltrating T cells in LLC-bearing mice treated with control, anti–PD-1 alone, cryoablation (CA) + PBS, or CA + anti–PD-1. B–C, Quantification of CD8⁺ (B) and CD4⁺ (C) T-cell densities across the four treatment arms. D–E, Representative images (D) and enumeration (E) of CD69⁺CD8⁺ activated cytotoxic T cells. F–G, Representative images (F) and counts (G) of IFN-γ⁺CD8⁺ effector T cells. H–I, Representative images (H) and quantification (I) of IFN-γ⁺CD4⁺ T cells. J and K, Flow-cytometry gating strategies for macrophages in tumor tissues (J) and PBMCs (K) from mice following macrophage depletion. L, Representative flow cytometry plots (left) and statistical analysis (right) of macrophage depletion in PBMCs. *P* values of B, C, E, J, and I were calculated using one-way ANOVA. The *p* value of L was calculated using a two-sided unpaired Student’s *t* test. Data are presented as mean ± SEM. ns, not significant; **p*<0.05, ***p*<0.01, ****p*<0.001. SEM, standard error of the mean.


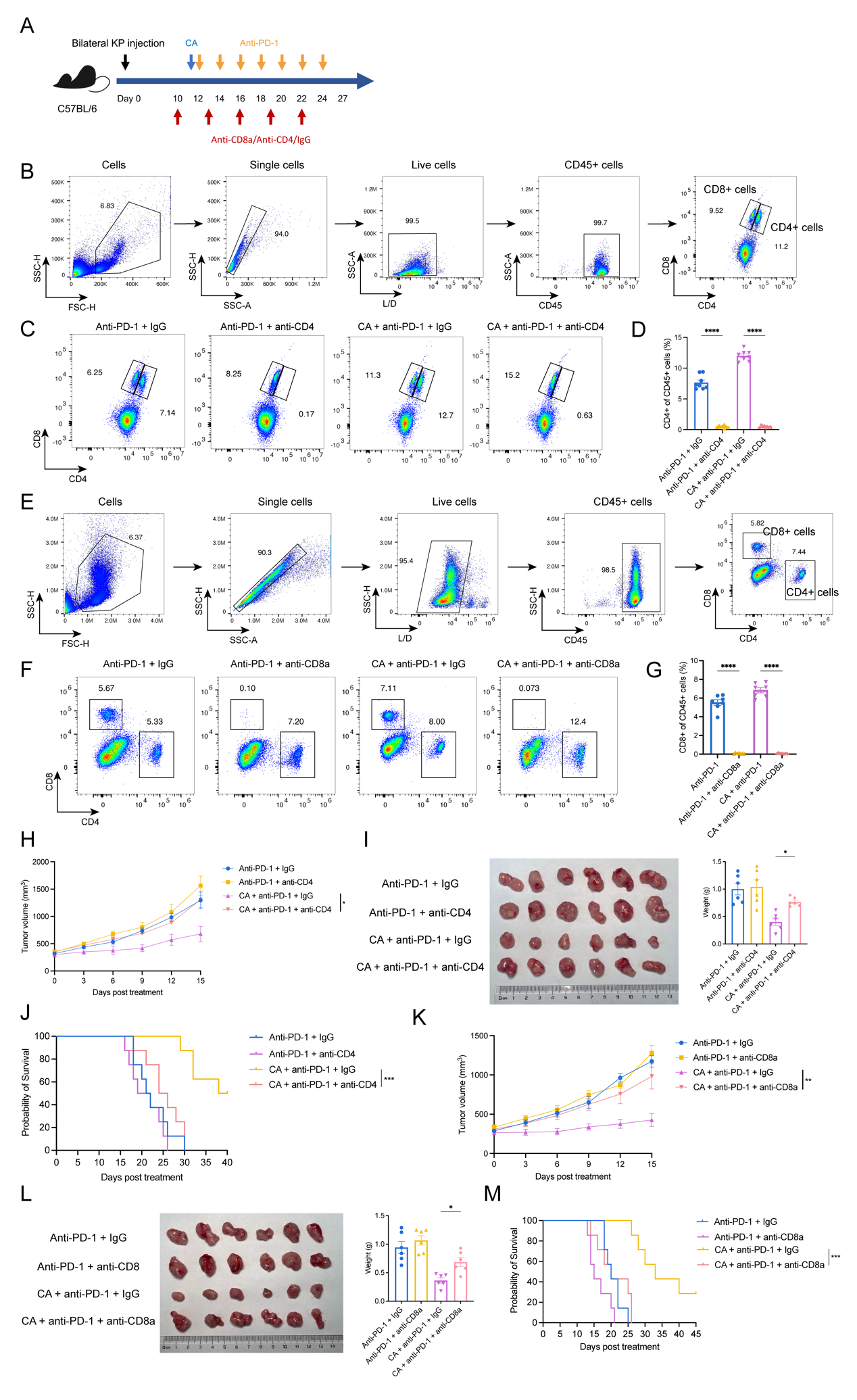


**Figure S5. In vivo CD4⁺ and CD8⁺ T-cell depletion in KP tumor bearing mice.**

A, Treatment schedule for CD4⁺ and CD8⁺ T-cell depletion in KP tumor–bearing mice. B, Flow-cytometry gating strategy for detecting CD4⁺ T cells in PBMCs of mice. C–D, Representative flow plots (C) and quantification (D) of CD4⁺ T cells in PBMCs after depletion. E, Flow-cytometry gating strategy for detecting CD8⁺ T cells in PBMCs of mice. F–G, Representative flow plots (F) and quantification (G) of CD8⁺ T cells in PBMCs after depletion. H-J. Tumor growth curves (H), tumor weight at day 15 post-treatment (I), and mouse survival curves (J) of the CD4+ T cell depletion experiment. K-M, Tumor growth curves (K), tumor weight (L), and mouse survival curves (M) of the CD8+ T cell depletion experiment.

D and H, n=8 mice/group; H, I, K and L, n=6 mice/group; G and M, n=7 mice/group. The *p* values of D, G, I and L were calculated using one-way ANOVA. *The p* values of J and M were determined by log-rank test (Mantel-Cox). Data are presented as mean ± SEM. **p*<0.05, ***p*<0.01, ****p*<0.001, *****p*<0.0001. SEM, standard error of the mean.


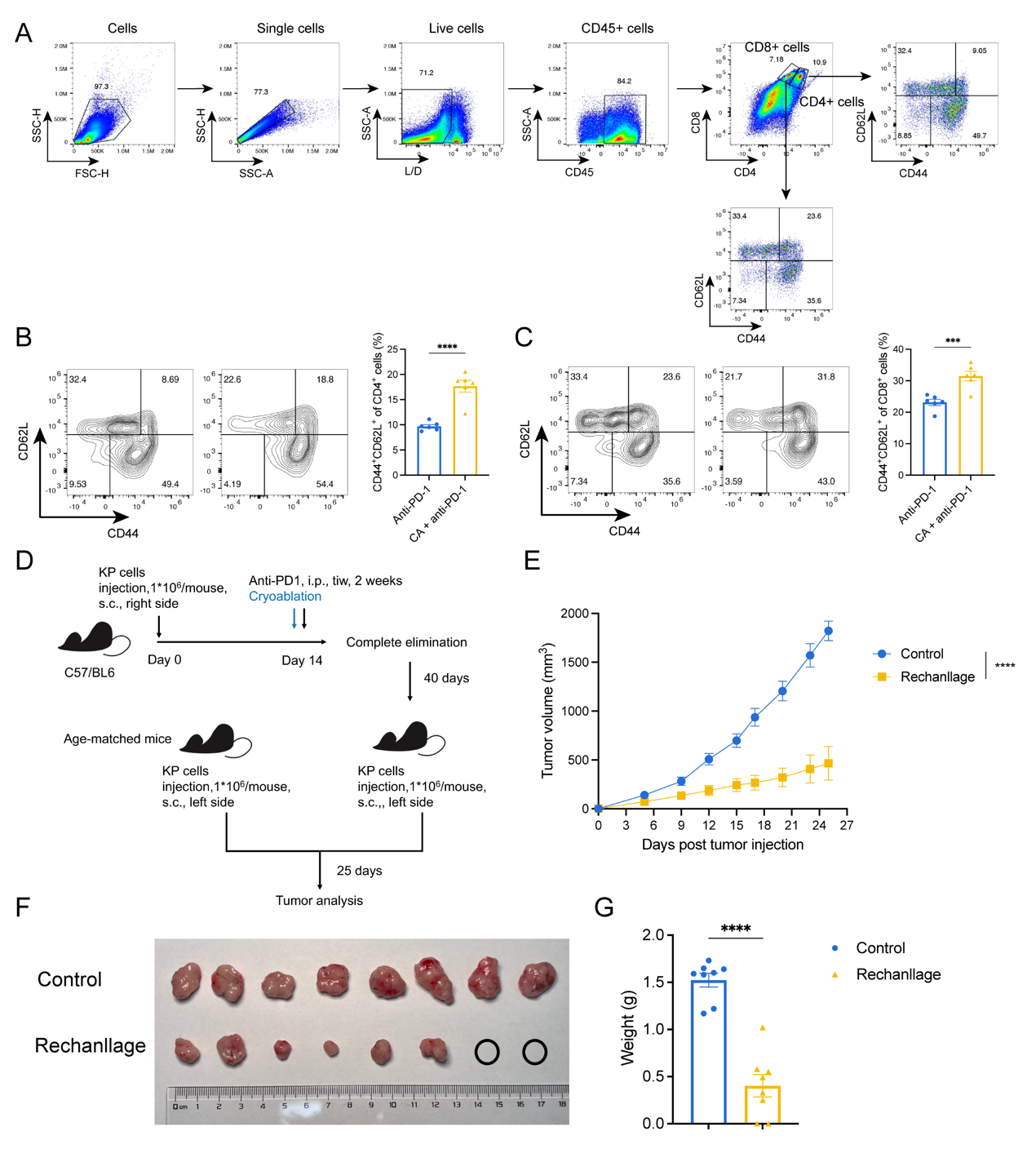


**Figure S6. Anti-tumor immune memory experiment.**

A, Flow cytometry gating strategy of spleen cells. B and C, Representative flow cytometry plots and quantification of splenic CD4⁺ TCM cells (B) and CD8⁺ TCM cells (C) in mice treated with cryoablation plus anti-PD-1 versus anti-PD-1 alone. D, Schematic diagram of the antitumor immune memory experiment. E–G, Tumor growth curves (E), representative tumor tissues (F), and tumor weights (G) in control and rechallenge groups. The *p* values of E and G were calculated using a two-sided unpaired Student’s *t* test. Data are presented as mean ± SEM. ****p*<0.001, *****p*<0.0001. SEM, standard error of the mean.


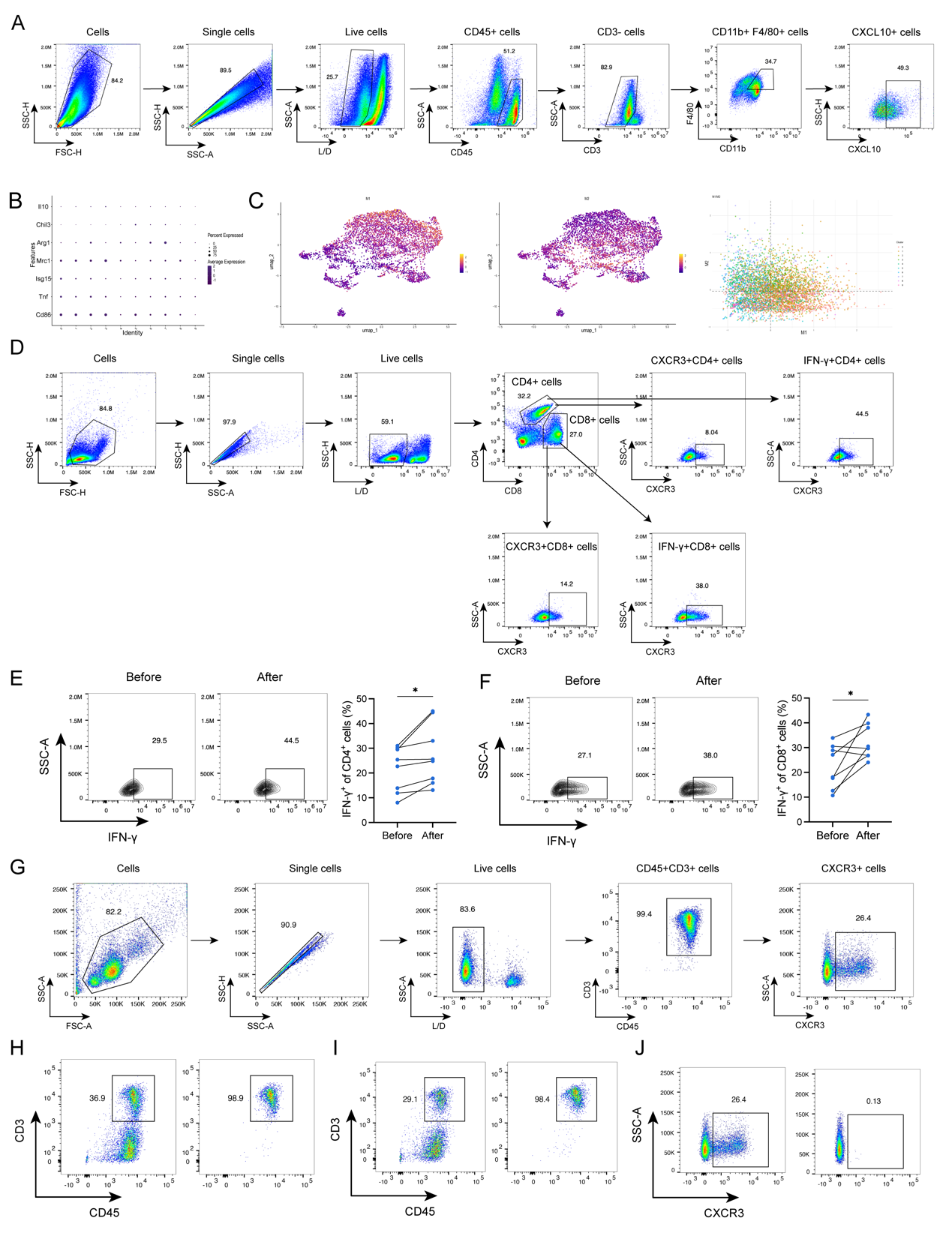


**Figure S7. Alterations of macrophage and T-cell subsets in the immune microenvironment following cryoablation**
A, Flow-cytometry gating strategy for CXCL10⁺ macrophages in mouse tumors. B, Bubble plot showing the expression levels of M1 and M2 marker genes in macrophage subtypes. C, UMAP plot demonstrating dimensionality reduction and clustering of macrophages based on M1 (left) and M2 (middle) marker gene expression levels. Two-dimensional projection displaying the expression levels of gene sets in individual cells (right). D, Gating scheme for CXCR3⁺CD4⁺ and CXCR3⁺CD8⁺ T cells in patient peripheral blood. E–F, Representative images and quantification of IFN-γ⁺CD4⁺ (E) and IFN-γ⁺CD8⁺ (F) T cells in PBMCs before versus after cryoablation in patients. G, Gating strategy for CXCR3+ T cells in mouse spleen; H and I, Sorting efficiency of splenic T cells from CXCR3 WT (H) and CXCR3 KO (I) mice. J, Flow cytometry validation of CXCR3 knockout efficiency in spleen of CXCR3 WT( left) and CXCR3-deficient mice (right). Data are presented as mean ± SEM. **p*<0.05. SEM, standard error of the mean.


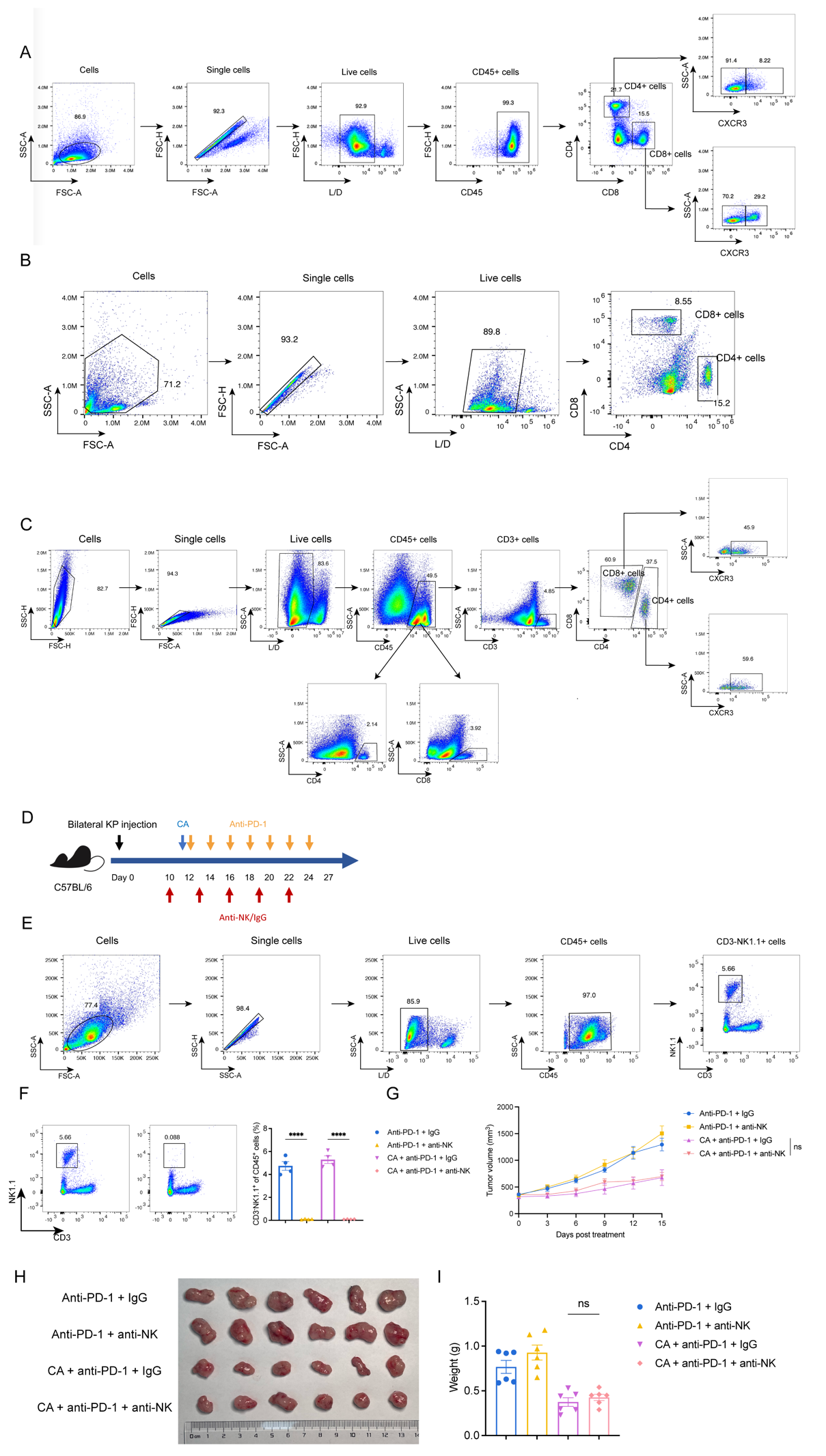


**Figure S8. Lymph node blockade and NK cell depletion experiment**

A–C, Flow-cytometry gating schemes for T-cell subsets in lymph nodes (A), PBMC (B), and tumor (C). **D**, Schematic diagram of NK cell depletion experiment. **E**, Gating strategy for mouse splenic NK cells. **F**, Representative flow cytometry plots (left) and statistical analysis (right) showing NK cell depletion efficiency. G- I, Tumor growth curves (G), representative images of tumors (H) and tumor weights (I) of the NK cells depletion experiment.


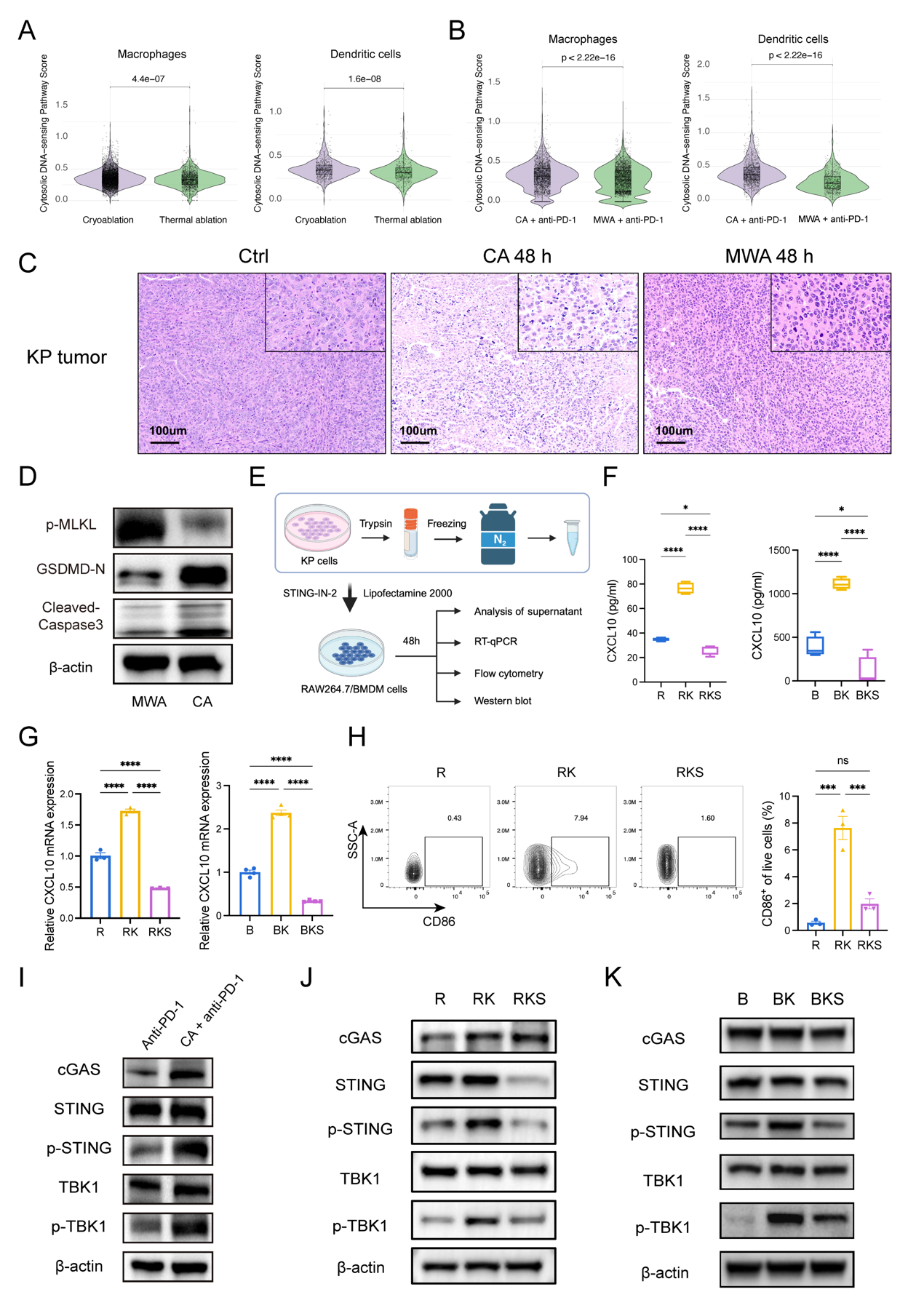


**Figure S9. Cryoablation activates the cGAS-STING signaling pathway in macrophages**

A, Cytosolic DNA-sensing pathway scores of macrophages (left) and DCs (right) in PBMCs from patients treated with cryoablation and thermal ablation. B, Cytosolic DNA-sensing pathway scores of macrophages (left) and DCs (right) in tumor tissues from mice treated with CA + anti-PD-1 and MWA + anti-PD-1. C, H&E staining of KP tumor tissues from control (left), cryoablation at 48 h (middle), and microwave ablation at 48 h (right). D, Western blot analysis of necrosis, apoptosis, and pyroptosis markers in ablated tumor tissues at 24 hours post-cryoablation and microwave ablation. E, Schematic diagram of the in vitro experimental setup showing lysis of KP cells by liquid nitrogen to activate RAW264.7 or BMDM. F, ELISA analysis of secreted CXCL10 in supernatants from RAW264.7 (left) and BMDM (right). G, qPCR analysis of CXCL10 mRNA expression in RAW264.7 (left) and BMDM (right) cells. H, Flow cytometry plots (left) and quantification (right) of CD86+ RAW264.7 cells. I, Western blot analysis of cGAS-STING pathway activation in non-ablated tumor tissues at 14 days post-cryoablation. J and K, Western blot analysis of cGAS–STING pathway proteins in RAW264.7 cells (K) and BMDMs (L) at 48 hours post-stimulation. Abbreviations: R, RAW264.7 cells; RK, RAW264.7 cells incubated with supernatant of KP cells lysed in liquid nitrogen; RKS, RAW264.7 cells incubated with supernatant of KP cells lysed in liquid nitrogen in the presence of STING-IN-2. B, BMDMs; BK, BMDMs co-cultured with supernatant of KP tumor cells lysed in liquid nitrogen; BKS, BMDMs co-cultured with supernatant of KP tumor cells lysed in liquid nitrogen in the presence of STING-IN-2.
